# The binding of Chp2’s chromodomain to methylated H3K9 is essential for Chp2’s role in heterochromatin assembly in fission yeast

**DOI:** 10.1371/journal.pone.0201101

**Published:** 2018-08-15

**Authors:** Vladimir Maksimov, Eriko Oya, Mayo Tanaka, Takayuki Kawaguchi, Aki Hachisuka, Karl Ekwall, Pernilla Bjerling, Jun-ichi Nakayama

**Affiliations:** 1 Department of Medical Biochemistry and Microbiology (IMBIM), Science for Life Laboratory, Uppsala University, Uppsala, Sweden; 2 Department of Biosciences and Nutrition, Karolinska Institutet, Huddinge, Sweden; 3 Division of Chromatin Regulation, National Institute for Basic Biology, Okazaki, Japan; 4 Department of Materials Science and Biotechnology, University of Fukui, Fukui, Japan; Tulane University Health Sciences Center, UNITED STATES

## Abstract

The binding of heterochromatin protein 1 (HP1) to lysine 9–methylated histone H3 (H3K9me) is an essential step in heterochromatin assembly. Chp2, an HP1-family protein in the fission yeast *Schizosaccharomyces pombe*, is required for heterochromatic silencing. Chp2 recruits SHREC, a multifunctional protein complex containing the nucleosome remodeler Mit1 and the histone deacetylase Clr3. Although the targeting of SHREC to chromatin is thought to occur via two distinct modules regulated by the SHREC components Chp2 and Clr2, it is not clear how Chp2’s chromatin binding regulates SHREC function. Here, we show that H3K9me binding by Chp2’s chromodomain (CD) is essential for Chp2’s silencing function and for SHREC’s targeting to chromatin. Cells expressing a Chp2 mutant with defective H3K9me binding (Chp2-W199A) have a silencing defect, with a phenotype similar to that of *chp2*-null cells. Genetic analysis using a synthetic silencing system revealed that a Chp2 mutant and SHREC-component mutants had similar phenotypes, suggesting that Chp2’s function also affects SHREC’s chromatin binding. Size-exclusion chromatography of native protein complexes showed that Chp2-CD’s binding of H3K9me3 ensures Clr3’s chromatin binding, and suggested that SHREC’s chromatin binding is mediated by separable functional modules. Interestingly, we found that the stability of the Chp2 protein depended on the Clr3 protein’s histone deacetylase activity. Our findings demonstrate that Chp2’s H3K9me binding is critical for SHREC function and that the two modules within the SHREC complex are interdependent.

## Introduction

Epigenetic changes affect genome function without an alteration of the DNA sequence, and they can be inherited by daughter cells and sometimes by offspring. Epigenetic changes in the genome are important in many diseases, including cancer, diabetes type II, and obesity [1,2]. Still, our knowledge about changes in the epigenome and the mechanisms that effect those changes is limited. In epigenetic regulation, various chromatin modifications confer diverse properties and functions to different types of chromatin. The basic unit of chromatin is the nucleosome, a complex between the DNA and the histone proteins [3]. The two basic types of chromatin are heterochromatin, which has low transcriptional activity, and euchromatin, which is actively transcribed [1,4]. Heterochromatin is characterized by low levels of histone acetylation and by the methylation of histone H3 lysine 9 (H3K9me), which provides a binding platform for chromodomain (CD) proteins such as the heterochromatin protein 1 (HP1)-family proteins [5,6].

The fission yeast *Schizosaccharomyces pombe* (*S*. *pombe*) is an excellent model system for studying the molecular mechanisms behind the transitions from one type of chromatin to another [5]. Several locations in the genome of fission yeast—the pericentromeres, the telomeres, and the mating-type region—are heterochromatic, and genes introduced into these regions are generally silenced. Heterochromatin formation and maintenance requires multiple steps; key among these are histone-tail deacetylation by deacetylases (Clr3, Clr6, and Sir2 in *S*. *pombe*) and the dimethylation or trimethylation of H3K9 (H3K9me) by methyltransferases (Clr4 in *S*. *pombe*), which provides a physical platform for HP1-family proteins [7–9]. *S*. *pombe* cells express two HP1 proteins, Swi6 and Chp2, and CDs in these proteins bind H3K9me *in vitro* with similar affinities [5,9,10]. The histone deacetylase Clr3 acts specifically on histone H3K14ac [7,11]. A fraction of the Clr3 protein joins the Snf2-related chromatin-remodeling factor Mit1, the Zn-finger-containing protein Clr1, the CD protein Chp2, and the Clr2 protein to form the SHREC complex [12,13]. Recent studies show that the SHREC complex can be divided into two distinct functional modules held together by the Clr1 protein: a remodeling module consisting of Mit1 and Chp2, which is thought to target chromatin via the Chp2-CD, and a histone deacetylase (HDAC) module consisting of Clr3 and Clr2, which is thought to target chromatin via a newly identified DNA/RNA-binding domain in Clr2 [12,14].

Here, we used genetic and biochemical approaches to examine Chp2’s role in heterochromatin assembly and in the SHREC complex in particular, and thereby demonstrated that Chp2’s binding to H3K9me is essential for its function in heterochromatin assembly in fission yeast. We also show that Chp2’s H3K9me binding is required for Clr3 to bind chromatin, and that Clr1 is important to the integrity of the SHREC complex. Surprisingly, we also found that Clr3’s HDAC activity was necessary for not only its own stability, but also that of Chp2. These results confirm SHREC’s modular chromatin targeting and reveal previously unidentified interactions between SHREC components.

## Materials and methods

### Constructs, strains, and media

Media was prepared as previously described [7]. To construct the plasmid for producing recombinant Chp2-CD proteins in *Escherichia coli* (*E*. *coli*) cells, the coding sequence was amplified by PCR and cloned into the pCRII vector with the TOPO-TA Cloning Kit (Invitrogen). After sequencing, the PCR product was subcloned into the pCold I vector (TaKaRa). To express Chp2-CD with the W199A mutation (Chp2-CD-W199A), the resultant plasmid was subjected to site-directed mutagenesis as described previously [15]. To obtain strains expressing FLAG-tagged Chp2, a portion of the *chp2*^+^-coding region (between –515 and +1700) was PCR-amplified and cloned into a pUC-derived plasmid with an *ura4*^+^ marker gene. Site-directed mutagenesis was used to introduce a *Bam*HI restriction site immediately after the ATG codon, and the DNA fragment for a 4× FLAG epitope was inserted into the *Bam*HI site. The W199A mutation was introduced by site-directed mutagenesis. The resultant plasmids were cleaved by *Bgl*II and introduced into the original *chp2*^+^ locus. To replace the wild-type *chp2*^+^ allele with the mutant *chp2* allele, strains that lost the *ura4*^+^ gene through internal homologous recombination were isolated using a counter-selective medium containing 5-fluoroorotic acid (FOA). All strains used in this study are listed in S1 Table.

### Recombinant protein production

The recombinant 6×His-tagged proteins used in isothermal titration calorimetry (ITC) analyses were expressed in *E*. *coli* BL21 (DE3) and purified by TALON Metal Affinity Resin (Clontech) according to the manufacturer’s instructions. Recombinant proteins were further purified by anion-exchange chromatography (Source 15Q; GE Healthcare).

### ITC

ITC was conducted using a MicroCal VP-ITC calorimeter (GE Healthcare) at 20°C. Recombinant Chp2-CD proteins were dissolved in phosphate buffer (20 mM KPO_4_ [pH 6.8] and 100 mM NaCl). A typical titration consisted of injecting 1-μl aliquots of ligand (H3K9me3; ARTKQTAR(Lys[Me]3)STGGKAPRY) into the protein sample at 3-min intervals to ensure that the titration peak returned to baseline. ITC data were analyzed using the Origin program.

### Silencing assays and images of yeast colonies

Spot tests were performed as previously described [16] using the following procedure: mid-log-phase cultures were adjusted to 3.2 × 10^6^ cells/ml, serially diluted in five steps, and applied to plates in 5-μl drops. A Canon EOS 1100D with a Canon EF 50-mm lens or MP-E 65 mm f/2.8 1–5× Macro Lens objective was used to take pictures of the yeast colonies.

### Real-time quantitative reverse transcription PCR (RT-qPCR)

*S*. *pombe* strains were grown in minimal medium (EMM) as shaken liquid cultures at 200 rpm, at 30°C, until log phase (1 × 10^7^ cells/ml). From each culture, 2 × 10^7^ cells were harvested by centrifugation at 3,000 ×*g* for 3 min at 4°C. Total yeast RNA was isolated with the RNeasy Mini Kit (Qiagen) according to the manufacturer’s instructions. RNA quality and concentrations were determined using 1% agarose gel electrophoresis and the NanoDrop^™^ 1000 spectrophotometer (Thermo Scientific). cDNA was synthesized with the Maxima First Strand cDNA Synthesis Kit for RT-qPCR (Thermo Scientific). Experiments were done in biological triplicates, with technical duplicates for each biological sample. For RT-qPCR, we used an MJ Mini^™^ Thermal Cycler (Bio-Rad) or a MiniOpticon^™^ Real-Time PCR System (Bio-Rad) with SYBR Green technology and the 5 × HOT FIREPol^®^ EvaGreen^®^ qPCR Supermix (Solis BioDyne). We used the following primers: ura4-Fw, 5’-CGTGGTCTCTTGCTTTTG-3’, ura4-Rv, 5’-GTAGTCGCTTTGAAGGTTAGG-3’; act1-Fw, 5’-GGTTTCGCTGGAGATGATG-3’, act1-Rv, 5’-ATACCACGCTTGCTTTGAG-3’ [17]. Data were presented as the fold enrichment in *ura4*^+^ transcript levels relative to the wild-type strain. Briefly, the threshold-cycle (*C*_*T*_) values were used to calculate Mean Normalized Expression (MNE) values, which were converted into fold-change values and normalized to gene expression in the wild-type strain [18]. Error bars indicated standard error of the mean (S.E.M.).

### Chromatin immunoprecipitation (ChIP) assay

The ChIP assay was modified from a previously described protocol [19,20]. Yeast cells were grown at 30°C until log phase (1 × 10^7^ cells/ml) in EMM shaken at 200 rpm. Cells (2 × 10^8^) were fixed with 1% formaldehyde (Merck) for 30 min at room temperature with gentle shaking at 120 rpm. Glycine was added to the cells to a final concentration of 125 mM while shaking gently for 5 min. Cells were collected by centrifugation at 3,000 ×*g*, for 5 min at 4°C and subsequently washed three times with 40 ml ice-cold phosphate-buffered saline (PBS). Pelleted cells were resuspended in 400 μl ice-cold lysis buffer (50 mM HEPES-KOH [pH 7.5], 150 mM NaCl, 1 mM EDTA, 1% Triton X-100, 0.1% Na-deoxycholate [DOC], 1 mM phenylmethylsulfonyl fluoride [PMSF], and 1× protease inhibitor cocktail [Complete^™^ EDTA-free; Roche]). Cell suspensions were combined with 500 μl of ice-cold glass beads (0.5-mm diameter, BioSpec Products) in 2-ml skirted micro tubes (Sarstedt) and lysed four times for 20 sec with FastPrep^®^ FP120 Cell Disrupter (BIO 101/Savant). Cell lysates were collected in 2-ml DNA LoBind tubes (Eppendorf), resuspended, and transferred to 1.5-ml DNA LoBind tubes (Eppendorf) before sonication using a Bioruptor^™^ Next Gen (Diagenode) at 4°C for 10 cycles, 30 sec on/60 sec off, with the power set to high. The samples were centrifuged at 16,000 ×*g* for 5 min at 4°C. The supernatant, which contained the soluble chromatin, was transferred to 1.5-ml DNA LoBind tubes. The pellet was resuspended in 400 μl of ice-cold lysis buffer and sonicated again, and the supernatants were pooled after centrifugation. The pooled chromatin samples were centrifuged one more time at 16,000 ×*g* for 10 min at 4°C, and the lysates (~800 μl) were placed in new 1.5-ml DNA LoBind tubes and kept on ice. DNA concentrations were measured with a NanoDrop^™^ instrument (Thermo Fisher Scientific).

In the ChIP assays, we used an anti-FLAG (F1804, Sigma-Aldrich) or anti-Myc (MA1-980, Thermo Fisher Scientific) antibody and Pierce ChIP-grade protein-A/G magnetic beads (Thermo Scientific/Pierce). Usually, each immunoprecipitation used 50–100 μg of lysate (50 μg of chromatin) and 20 μl of beads (50% slurry) bound to 2 μg of antibody, and total volumes were adjusted to 250 μl with the lysis buffer. Unbound magnetic beads were used for control immunoprecipitations. Input fractions (10%) were removed from the chromatin lysates prior to immunoprecipitation, adjusted to the total volumes of 250 μl with 50 mM Tris-HCl (pH 8.0), 10 mM EDTA, and 1% SDS, incubated overnight at 65°C, cooled to room temperature, and treated with DNase-free RNase A (Thermo Fisher Scientific) for 1 h at 37°C and Proteinase K (Thermo Fisher Scientific) for 3 h at 56°C. The immunoprecipitated chromatin beads (bound fraction) and control (unbound, no antibody) beads were washed twice with lysis buffer, once with wash buffer I (10 mM Tris-HCl [pH 8.0], 250 mM LiCl, 0.5% NP-40, 0.5% DOC, and 1 mM EDTA), and once with TE buffer (10 mM Tris-HCl [pH 8.0] and 1 mM EDTA) containing 0.05% Tween 20. DNA was extracted twice using TES elution buffer (50 mM Tris-HCl [pH 8.0], 10 mM EDTA, and 1% SDS) for 30 min at 65°C. The elution fractions were mixed, incubated overnight at 65°C, and treated as described for input fractions. DNA was extracted from the solutions using the QIAquick PCR Purification Kit (Qiagen) or MiniElute PCR Purification Kit (Qiagen).

To amplify immunoprecipitated *ura4*^+^, *act1*^+^, and centromeric *dg* DNA sequences, real-time qPCR was performed in duplicates for each technical replicate using the same primers as for RT-qPCR. Relative DNA quantification was carried out at least for two biological replicates and technical duplicates for each biological replica. Real-time PCR data were analyzed by the percent input method [18]. Briefly, the *C*_*T*_ values for input and bound (IP) fractions were averaged. The input DNA *C*_*T*_ values for the *ura4*^+^ and *act1*^+^ genes were adjusted from 10% (starting input fraction) to the 100% equivalent by subtracting 3.32 *C*_*T*_ (Log_2_10). The percent input was calculated by 100 x AE^(*C*_*T*_ [adjusted Input]–*C*_*T*_ [IP]), where AE is the amplification efficiency for a given pair of primers. We normalized the % input values for the *ura4*^+^ and *act1*^+^ genes relative to the wild-type strain; data were represented as the relative fold-change in % input for the corresponding gene, with error bars indicating the standard deviation (SD).

### Native yeast-cell protein extracts

Yeast cells were grown at 30°C to late log-phase (1.5 × 10^7^ cells/ml) in 100 ml of rich-growth YEA medium shaken at 200 rpm. Cells were harvested by centrifugation at 3,000 ×*g* for 3 min at 4°C, washed twice with 40 ml of ice-cold 1× stop buffer (150 mM NaCl, 50 mM NaF, 10 mM EDTA, and 1 mM NaN_3_), and finally transferred into 3 × 2-ml micro tubes (Sarstedt) and centrifuged at 3,000 ×*g* for 5 min at 4°C to remove the stop buffer. The pelleted cells were combined with 750 μl of ice-cold glass beads (0.5-mm diameter, BioSpec Products) along with 400 μl of ice-cold native lysis buffer (50 mM Tris-HCl [pH 8.0], 150 mM NaCl, 50 mM NaF, 10% glycerol, 2 mM EDTA, 10 mM EGTA, 0.5% Triton X-100, 0.5% IGEPAL^®^ CA-630 [Sigma-Aldrich], 5 mM ß-glycerophosphate, 0.1 mM Na_3_VO_4_, 1 mM ß-mercaptoethanol, 2 mM dithiothreitol [DTT], 1 mM PMSF, and 2.5 × protease inhibitor cocktail [Complete^™^ EDTA-free; Roche]) and were lysed twice for 20 sec at 4°C with a FastPrep^®^ FP120 Cell Disrupter (BIO 101/Savant) on maximum power. An additional 400 μl of ice-cold native lysis buffer was added to the samples, and the crude native lysates were incubated for 15 min in a rotating wheel at 4°C (for RNase A treatment, DNase- and protease-free RNase A [Thermo Scientific] was added to the native lysis buffer to a final concentration of 1 mg/ml). After incubation, the lysate was transferred to DNA LoBind tubes (Eppendorf), and the samples were centrifuged for 10 min at 16,000 ×*g* at 4°C. The supernatants were carefully transferred to a glass beaker, mixed together, filtered with a 0.20-μm filter unit (Sarstedt), and kept on ice before loading into columns for size-exclusion chromatography. The protein concentration in the lysates, measured with a NanoDrop^™^ 1000 (Thermo Fisher Scientific), was typically 20–25 mg/ml.

### Gel filtration chromatography

Native protein extracts were fractionated by fast protein liquid chromatography (FPLC). Briefly, 1 ml of pre-cleared native protein extract was loaded onto a HiLoad 16/600 Superdex 200 prep-grade column (GE Healthcare) attached to an ÄKTAxpress FPLC system (GE Healthcare) and equilibrated with running buffer (50 mM Tris-HCl [pH 7.5], 150 mM NaCl, 0.05% Tween 20, and 2 mM DTT). The column was also calibrated with a Gel Filtration HMW Calibration Kit (GE Healthcare) at running conditions. The proteins were fractionated at 4°C with a flow rate of 1 ml/min. After discarding the first 30-ml flow-through, 2-ml fractions were collected, and the proteins were precipitated according to the Peterson trichloroacetic acid (TCA)-DOC protocol with some modifications. Briefly, a 1/10 volume of 0.15% DOC solution was added to the fraction sample and mixed by inverting the tube several times, and the sample was incubated on ice for 15 min. Next, 1/10 of the original sample volume of chilled 72% TCA was added, and the sample was mixed by inverting the tube several times, and then incubated on ice for 15 min. The samples were centrifuged at 16,000 ×*g* for 15 min at 4°C and the supernatant carefully discarded. Pre-chilled (–20°C) acetone containing 10 mM ß-mercaptoethanol was added to the samples and the tubes were vortexed for 30 sec. The samples were left overnight at –20°C, then centrifuged at 16,000 ×*g* for 15 min at 4°C. The supernatants were discarded, and the pellets were air-dried at room temperature for 5–10 min and solubilized with 30 μl Urea-SDS buffer (50 mM Tris-HCl [pH 8.0], 8 M urea, 1% SDS, 10 mM ß-mercaptoethanol, and 10 mM DTT).

### Trichostatin A (TSA) treatment

*S*. *pombe* strain PJ1911 (*V5-clr2 clr3-myc FLAG-chp2*) was grown in 10 ml of rich YEA medium containing 1% ethanol or 5–10 μg/μl trichostatin A (Sigma-Aldrich, dissolved in 99% ethanol), from 2.5 × 10^6^ cells/ml to the log phase (1 × 10^7^ cells/ml). Cells were collected by centrifugation at 3,000 ×*g* for 5 min at 4°C, resuspended in 1 ml ice-cold stop buffer (150 mM NaCl, 50 mM NaF, 10 mM EDTA, and 1 mM NaN_3_), transferred into 2-ml skirted micro tubes (Sarstedt), and centrifuged at 3,000 ×*g* for 5 min at 4°C to remove the stop buffer. Cell pellets were resuspended in 100 μl of ice-cold RIPA buffer (50 mM Tris-HCl [pH 8.0], 150 mM NaCl, 1% Triton X-100, 0.1% SDS, 2 mM EDTA, 50 mM NaF, 0.1 mM Na_3_VO_4_, 5 mM ß-glycerophosphate, 20 mM ß-mercaptoethanol, and 1× protease inhibitor cocktail [Complete^™^ EDTA-free, Roche]), boiled for 6 min at 98°C, cooled on ice, and frozen in liquid nitrogen. Whole-cell extracts were prepared using 400 μl of glass beads (0.5-mm diameter, BioSpec Products) and a FastPrep^®^ FP120 Cell Disrupter (BIO 101/Savant) on maximum power for 30 sec at 4°C. After bead beating, 200 μl of protein-extraction buffer (50 mM Tris-HCl, [pH 8.0], 3% SDS, 2 mM EDTA, and 20 mM ß-mercaptoethanol) was added to each sample (to a final concentration of 1% SDS) and mixed thoroughly by vortexing. Samples were boiled for 10 min at 98°C and centrifuged at room temperature for 10 min at 16,000 ×*g*. Supernatants were carefully transferred to the 1.5-ml Eppendorf tubes, and 2-μl aliquots were taken out and measured for protein concentration with a NanoDrop^™^ 1000 (Thermo Fisher Scientific).

### SDS-PAGE and western blotting

Protein extracts (200 μg) or recovered proteins from FPLC fractions (15 μl) were loaded onto 8% SDS-PAGE gel and run at 125 V for 80 min. The proteins were transferred to PVDF membranes (Thermo Fisher Scientific) by the wet-transfer method with transfer buffer (25 mM Tris, 192 mM glycine, 10% methanol, and 0.005% SDS) at 20 mA for 60 min at 4°C. The membranes were blocked with 5% skim milk in 1× PBST buffer (1× PBS containing 0.05% Tween 20). The following primary antibodies were used for immunoblotting: anti-V5 tag (mouse, R960-25, Invitrogen), anti-FLAG (rabbit, F7425, Sigma-Aldrich), anti-c-Myc (9E10) (mouse, 13–2500, Invitrogen), anti-ß-actin (mouse, ab8224, Abcam), and anti-histone H3 C-terminus (rabbit, ab1791, Abcam), all diluted 1:7,500 in 1× PBST buffer, and anti-Chp2 (rabbit [10]) diluted 1:1,000 in 1× TBST buffer (1× TBS containing 0.05% Tween 20). As secondary antibodies, anti-mouse IgG HRP-linked whole Ab (GE Healthcare) and anti-rabbit IgG, HRP-linked whole Ab (GE Healthcare), diluted 1:7,500 in 1× PBST buffer, were used. Membranes were incubated with Immobilon Western Chemiluminescent HRP Substrate (WBKLS0500, Millipore), exposed from 5 sec to 10 min with a ChemiDoc^™^ MP Imaging System (Bio-Rad), and analyzed with Image Lab^™^ software (Bio-Rad).

## Results

### A conserved tryptophan residue in Chp2’s chromodomain is necessary for binding H3K9me

**Fig 1:**
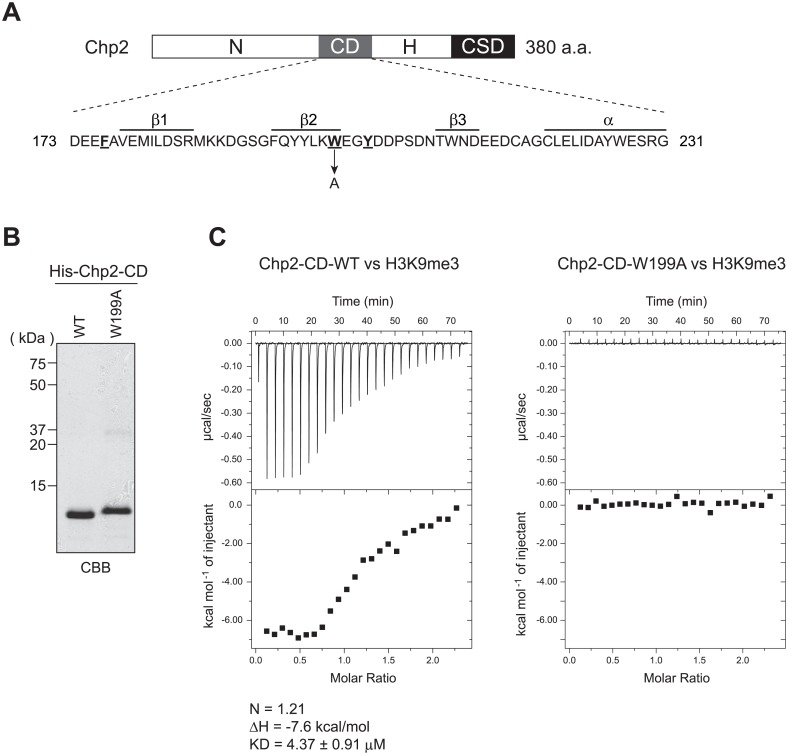
The Chp2 chromodomain binds H3K9me. (A) Schematic of the Chp2 protein showing the amino acid sequence of the CD domain and the W199A mutation. N: N-terminal domain; CD: chromodomain; H: hinge region; CSD: chromoshadow domain. (B) Recombinant proteins used in (C) were resolved by SDS-PAGE and visualized by Coomassie brilliant blue (CBB) staining. (C) Isothermal titration calorimetry (ITC) results for Chp2-CD (left) and Chp2-CD-W199A (right). The raw heat data were obtained upon the injection of H3K9me3 peptide into solutions of each protein. The heat data were integrated with respect to time, and injection enthalpy was determined from the best-fit curve. The binding constant (K_D_), enthalpy change (ΔH), and stoichiometry (N) values are indicated below each graph. These values were not determined for Chp2-CD-W199A due to its weak binding of the H3K9me3 peptide.

HP1-family proteins recognize histone H3 methylated on lysine 9 (H3K9me) via their CD [6,9], which consists of four ß-strands and an α-helix and it recognizes H3K9me through an aromatic cage formed by three conserved residues (Fig 1A, underlined) [6]. To examine Chp2’s function in targeting SHREC to chromatin, we first characterized Chp2-CD’s binding to H3K9me. We purified recombinantly expressed Chp2-CD (Fig 1B, WT) and tested its binding to K9-trimethylated histone H3 (H3K9me3) peptide (1–18). ITC showed that Chp2-CD bound the H3K9me3 peptide with an affinity of *K*_*D*_ = 4.37 ± 0.91 μM (Fig 1C, left panel), which agrees with previous reports [10]. To determine whether Chp2-CD binds the H3K9me3 peptide through its conserved hydrophobic residues, we created a mutant Chp2-CD protein (Chp2-CD-W199A, in which tryptophan 199, one of the three conserved residues, was changed to alanine) (Fig 1A and 1B; W199A), and tested its affinity for H3K9me3 peptide (Fig 1C, right panel). The W199A mutation clearly abolished Chp2-CD’s binding to the H3K9me3 peptide, since titrating the methylated peptide did not produce any calorimetrical change (Fig 1C). Thus, the conserved W199 residue in the Chp2-CD was necessary for Chp2 to bind H3K9me3. We used this W199A mutation to further analyze Chp2’s function.

### Chp2’s H3K9me binding is essential for its function in heterochromatic silencing

To investigate the role of H3K9me binding in Chp2’s function, we created a strain that expressed a mutated Chp2 protein containing W199A (Chp2-W199A) from the endogenous *chp2*^+^ locus (Fig 1A; see Materials and methods). Since the level of endogenous Chp2 expression is relatively low [10], we introduced multiple FLAG tags simultaneously into the Chp2 N-terminus to allow us to readily detect Chp2 (F-Chp2-W199A) (S1A Fig). As a control, we also created a strain expressing the wild-type Chp2 protein with the same FLAG tags from the endogenous promoter (F-Chp2) (S1A Fig).

**Fig 2:**
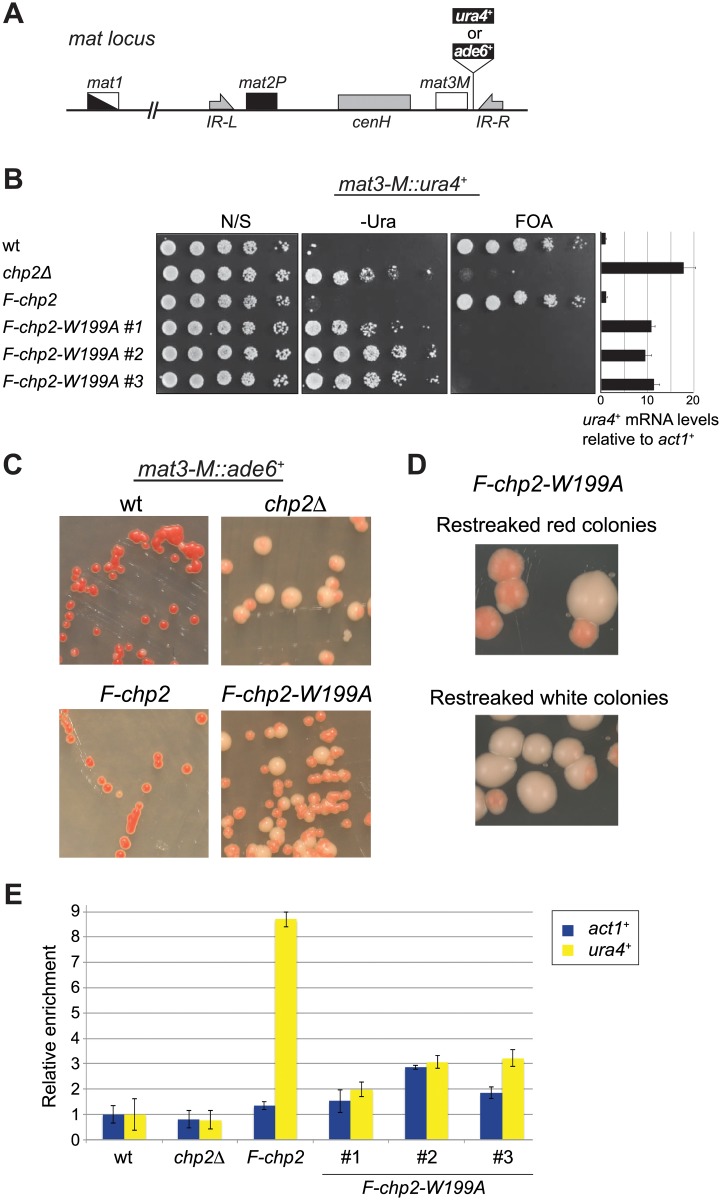
Chp2’s H3K9me-binding ability is essential for its function in heterochromatin formation. (A) Schematic of the mating-type (*mat*) locus, showing the position of the silencing-reporter gene (*mat3-M*::*ura4*^+^ or *mat3-M*::*ade6*^+^). Indicated in the picture are the mating-type cassettes–*mat1*, *mat2P*, and *mat3M* –as well as the *inverted repeat left* (*IR-L*), *inverted repeat right* (*IR-R*) and the region homologous to the pericentromeric region, *cenH*. The *mat* locus is not drawn to scale. (B) Cells were serially diluted in five steps and spotted onto non-selective medium (N/S), selective medium lacking uracil (–Ura), and counter-selective medium containing FOA. The amount of *ura4*^+^ transcript was quantified by RT-qPCR and normalized to *act1*^+^ and calculated as fold change to the wild-type expression. The strains used were PJ1811 (wt), PJ1813 (*chp2*Δ), PJ1815 (*F-chp2*), PJ1817, PJ1818, and PJ1819 (*F-chp2-W199A*). (C) Wild-type and mutant strains with an *ade6*^+^ reporter gene inserted into the mating-type region were streaked on medium containing low amount of adenine (YE medium). The strains used were PJ1044 (wt), PJ1569 (*chp2*Δ), PJ1770 (*F-chp2*), and PJ1538 (*F-chp2-W199A*) (D) Red or white colonies from (C) were restreaked on YE medium. (E) Chromatin immunoprecipitation (ChIP) assay showing relative enrichment of F-Chp2 signal at *mat3-M*::*ura4*^+^ and *act1*^+^. Data were normalized to wild-type (non-tag) strain’s signals at *mat3-M*::*ura4*^+^ and *act1*^+^. Error bars indicate standard deviation (SD). The strains used were FY597 (wt), PJ748 (*chp2*Δ), PJ1745 (*F-chp2*), PJ1735, PJ1736, and PJ1737 (*F-chp2-W199A*).

Next, we examined the impact of Chp2’s H3K9me binding on its function in heterochromatic silencing. The *F-chp2-W199A* allele or the control *F-chp2* was combined with the *mat3-M*::*ura4*^+^ reporter gene (Fig 2A), and the silencing states were evaluated by a spotting assay in which cells were cultured, serially diluted, and spotted onto either non-selective plates (N/S), plates to select for *ura4*^+^-expressing cells (–Ura), or counter-selective plates containing 5-fluoroorotic acid (FOA) (Fig 2B) [21]. The *ura4*^+^ gene inserted into the mating-type region was tightly repressed in wild-type cells, which grew poorly on–Ura plates but grew well on FOA plates (Fig 2B, wt). A lack of Chp2 derepressed the reporter gene, as evidenced by good cell growth on–Ura plates and no growth on plates containing FOA (Fig 2B, *chp2*Δ); this agrees with previously published data [10,12]. RT-qPCR also confirmed the derepression state of *mat3-M*::*ura4*^+^ (Fig 2B, right). We confirmed that adding the FLAG tags to Chp2’s N-terminus did not disturb its function, since the silencing state of the *mat3-M*::*ura4*^+^ reporter was comparable to that in wild-type cells (Fig 2B, *F-chp2*).

Cells expressing F-Chp2-W199A showed defective silencing in the spotting assay and in RT-qPCR (Fig 2B), although RT-qPCR revealed slightly milder derepression in *F-chp2-W199A* than in *chp2*Δ cells, with slight variations between independent isolates (Fig 2B, last three rows). There were two possible explanations for this partial derepression: either silencing was alleviated at a similarly low level in all cells, or the culture contained cells with variegated derepression states [16,22]. To distinguish between these possibilities, we used a *mat3-M*::*ade6*^+^ allele, in which the *ade6*^+^ gene was inserted into the same position as the *ura4*^+^ reporter (Fig 2A). When wild-type cells repressing *mat3-M*::*ade6*^+^ were plated on a medium containing limited amounts of adenine, the cells formed red colonies due to the accumulation of an intermediate in the adenine-synthesis pathway (Fig 2C, top left) [23]. A control strain expressing the F-Chp2 protein also formed red colonies on the plates, like the wild-type strain (Fig 2C, bottom left), while the *chp2*Δ and *F-chp2-W199A* strains formed two types of colonies—smaller pink and larger white colonies (Fig 2C, right panels). The existence of two cell populations in these mutant strains indicated that there was indeed an epigenetic shift between the repressed and derepressed chromatin states at the mating-type region. However, repressed colonies were light pink rather than red indicating that the *ade6*^+^-reporter gene was not fully repressed, suggesting that both *chp2* mutations caused a partial derepression in all cells and full derepression in a certain percentage of the cells.

To confirm that the two mutant strains had similar phenotypes, we restreaked pink and white colonies and counted how many pink versus white colonies were generated in the offspring (Fig 2D and Table 1). A restreaked pink colony formed by the *chp2*Δ strain generated 78% pink and 22% white new colonies; the *chp2-W199A* strain formed 79% pink and 21% white new colonies. This very similar frequency of switching indicates that the two mutations had very similar effects on mating-type silencing.

**Table 1:** Epigenetic switching between different expression states.

Genotype	Original colony color	Number of red colonies (percentage)	Number of white colonies (percentage)
**Wild-type**	Red	66 (100)	0 (0)
	White	ND	ND
***chp2*Δ**	Red	80 (78)	23 (22)
	White	25 (33)	50 (67)
***F-chp2***	Red	57 (100)	0 (0)
	White	ND	ND
***F-chp2-W199A***	Red	174 (79)	46 (21)
	White	49 (34)	97 (66)

Red and white colonies were picked and restreaked on fresh YE plates. After 3 days at 30°C, the number of colonies of each color was counted. ND, not detected.

To determine whether the *chp2-W199A* mutant’s phenotype was due to a reduced binding of Chp2-W199A to the mating-type region, we conducted ChIP assays with an anti-FLAG antibody and primers against the *act1*^+^ (control) and *ura4*^+^ genes. The strain expressing wild-type F-Chp2 protein was strongly enriched at the *mat3-M*::*ura4*^+^ region (Fig 2E, *F-chp2*), whereas three different isolates of the strains expressing F-Chp2-W199A were only slightly or moderately enriched in F-Chp2-W199A bound to the *mat3-M*::*ura4*^+^ gene. The enrichment of F-Chp2-W199A bound to the *act1*^+^ control gene was slightly higher than that of the wild-type strain, which may indicate a promiscuous association of F-Chp2-W199A with euchromatic genes (Fig 2E; compare *F-chp2* to *F-chp2-W199A*). Taken together, these results indicated that Chp2’s ability to bind H3K9me is crucial for its silencing function.

### Chp2’s H3K9me binding is necessary for targeted silencing

**Fig 3:**
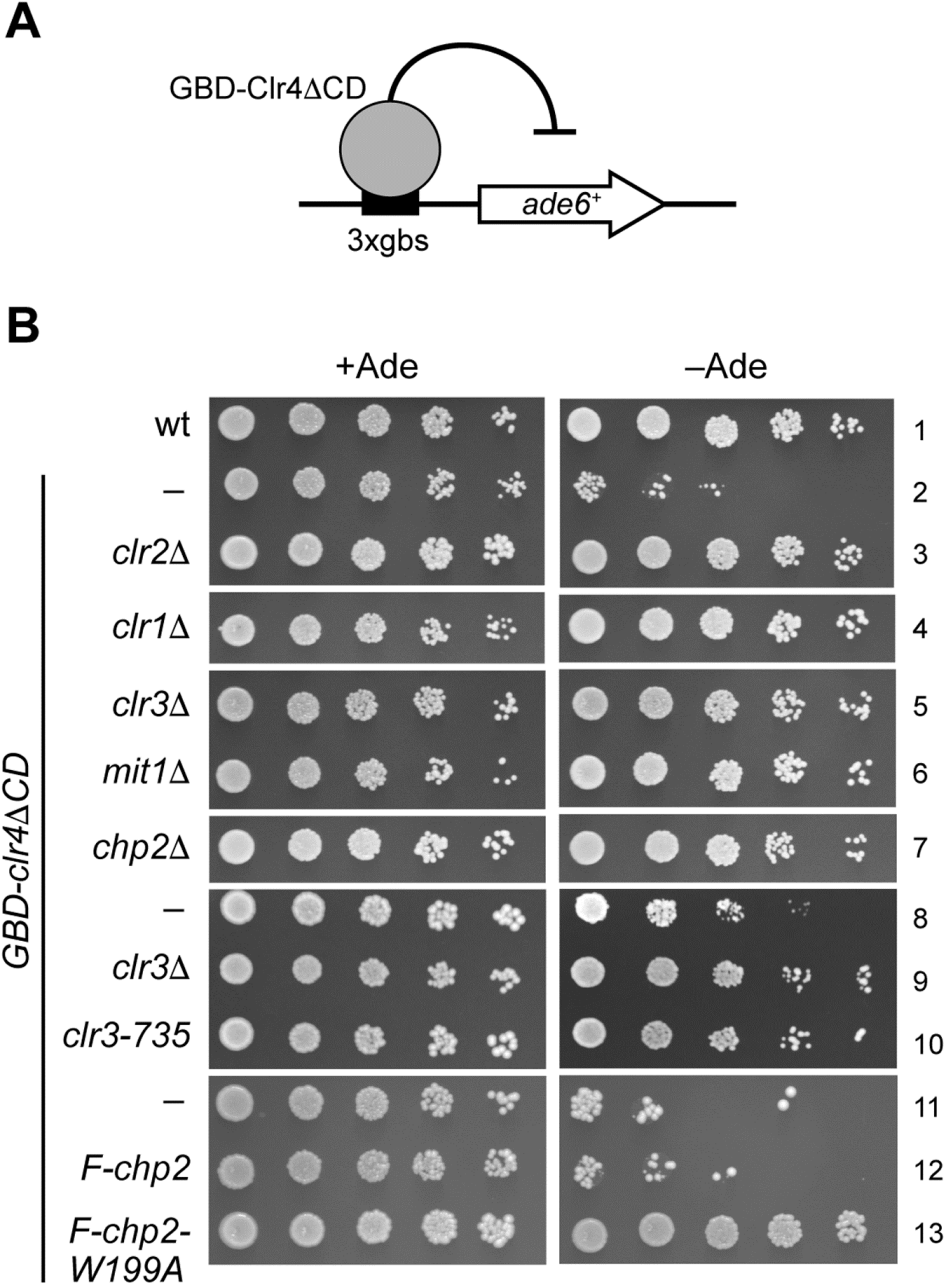
Chp2’s H3K9me binding is required for targeted silencing by GBD-Clr4ΔCD. (A) Schematic showing the targeted silencing assay, in which a Gal4 DNA-binding domain (GBD)-fused Clr4 lacking its chromodomain (GBD-Clr4ΔCD) was tethered to 3× gal-binding sites (3×gbs) upstream of an *ade6*^+^-reporter gene. (B) Serially diluted cells were spotted onto non-selective (+Ade) or selective (–Ade) plates. The strains used were PJ1207 (wt), PJ1231 (*GBD-Clr4*Δ*CD*), PJ1327 (*GBD-Clr4*Δ*CD clr2*Δ), PJ1246 (*GBD-Clr4*Δ*CD clr1*Δ), PJ1247 (*GBD-Clr4*Δ*CD clr3*Δ), PJ1320 (*GBD-Clr4*Δ*CD mit1*Δ), PJ1314 (*GBD-Clr4*Δ*CD chp2*Δ), PJ1318 (*GBD-Clr4*Δ*CD*), PJ1316 (*GBD-Clr4*Δ*CD clr3-735*), PJ1325 (*GBD-Clr4*Δ*CD F-chp2*), and PJ1331 (*GBD-Clr4*Δ*CD F-chp2-W199A*).

Having established that Chp2’s molecular function is to bind chromatin via H3K9me, we next focused on Chp2’s function in the SHREC complex. We used a minimal silencing system in which an engineered Clr4 protein lacking the CD and fused to a Gal4 DNA-binding domain (GBD-Clr4ΔCD) silences a reporter gene placed downstream of a triple Gal4-binding site (3×gbs) integrated at a euchromatic location (Fig 3A) [24]. In fission yeast, several distinct pathways cooperate to assemble repressive heterochromatin, making it difficult to define the exact role of trans-acting factors based on a reporter gene inserted into the mating-type region. However, a minimal silencing assay allows us to define factors that are specifically required for Clr4-mediated heterochromatin assembly. Previous studies using this system revealed distinct roles of the RNAi pathway and demonstrated that three SHREC components—Clr2, Clr3, and Chp2—are necessary for targeted silencing [22,24] (Fig 3B).

To examine the effect of Chp2’s H3K9me binding on SHREC’s function, we first tested whether the SHREC-complex components are individually required in targeted silencing. Serially diluted cells were spotted onto non-selective plates (+Ade) and plates without adenine (–Ade) to evaluate the silencing state of the *ade6*^+^-reporter gene (Fig 3B). As previously observed [22,24], cells expressing wild-type Clr4 protein did not silence the *ade6*^+^ gene, whereas cells expressing GBD-Clr4ΔCD repressed the reporter gene and grew poorly on plates lacking adenine (Fig 3B; compare the two first rows). Strains lacking any one of the SHREC components—Clr1, Clr2, Clr3, Mit1, or Chp2—expressed the *ade6*^+^ reporter and grew well on selective plates (Fig 3B, rows 3–7), suggesting that all components of the SHREC complex were necessary for targeted silencing. We also confirmed that Clr3’s HDAC activity was crucial for ectopic silencing; in a strain with a *clr3-735* loss-of-function mutation [25], this silencing was clearly absent (Fig 3B; compare rows 9 and 10). Importantly, the strain expressing the F-Chp2-W199A protein also failed to silence the reporter gene; it had a derepression state comparable to that of the SHREC-component mutants (Fig 3B, 13th row). These results confirmed that Chp2-CD’s H3K9me binding is important in targeted silencing, and indicated a functional link between Chp2 and SHREC’s targeting to chromatin.

### Chp2’s H3K9me binding affects Clr3’s chromatin targeting

SHREC was previously shown to contain two functional modules and to target chromatin through separable recruitment mechanisms [12,14]. To test whether Chp2’s H3K9me binding affects SHREC’s chromatin targeting, we combined yeast strains expressing myc-tagged Clr3 (Clr3-myc), V5-tagged Clr2 (V5-Clr2) [7,22], or F-Chp2 with each of the SHREC-component mutant alleles. The protein complexes in native whole-cell lysates of these strains were separated by gel-filtration chromatography (see Materials and methods). Proteins in the collected fractions were resolved by SDS-PAGE and analyzed by western blotting.

**Fig 4:**
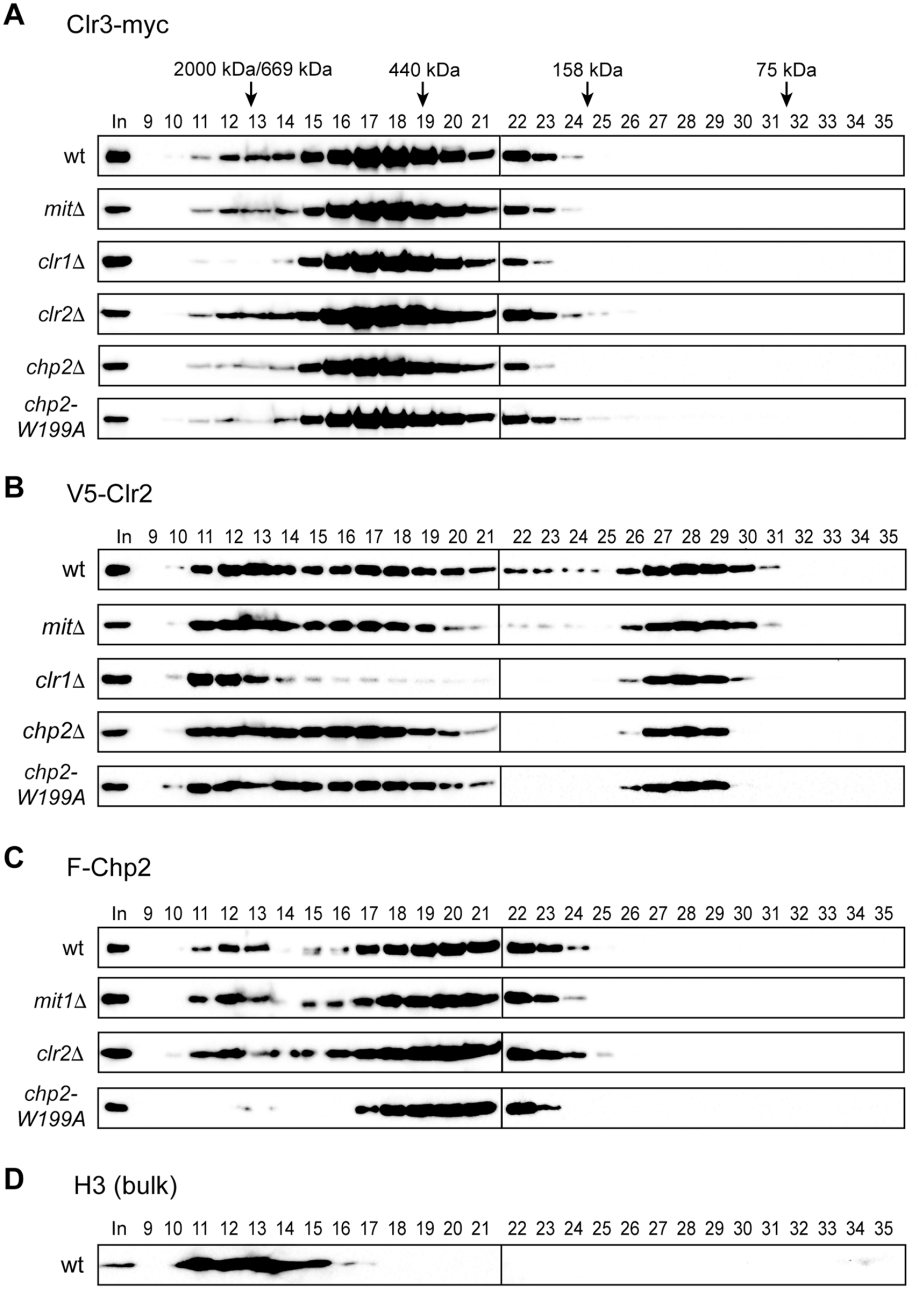
Chp2’s H3K9me binding affects Clr3’s chromatin targeting. (A–D) Native whole-cell lysates prepared from indicated strains were fractionated by gel-filtration chromatography. Proteins were precipitated from collected fractions and analyzed by 8% SDS-PAGE. Elution profiles of Clr3-myc (A), V5-Clr2 (B), F-Chp2 (C) and bulk H3 (D) were analyzed by western blotting. The first band in each panel represents the 1% input from yeast lysates (In). The strains used were PJ1911 (*V5-clr2 clr3-myc F-chp2*), PJ2012 (*mit1*Δ *V5-clr2 clr3-myc F-chp2*), PJ1836 (*clr1*Δ *V5-clr2 clr3-myc*), PJ1994 (*clr2*Δ *clr3-myc F-chp2*), PJ1840 (*chp2*Δ V*5-clr2 clr3-myc*), and PJ1917 (*V5-clr2 clr3-myc FLAG-chp2W199A*).

Clr3-myc has an expected molecular mass of 92.4 kDa, and structural analysis suggests that Clr3 forms a dimer [14]. Clr3-myc in the native lysate was detected in fractions 11–24 with at least two peaks: Most of the Clr3-myc was eluted in fractions 16–19, corresponding to ~500 kDa, whereas a minor Clr3-myc population was detected near the void fractions 11–14 (>600 kDa) (Fig 4A, wt). Although the molecular mass and stoichiometry of the native SHREC complex is not clear, the faster-eluted peak probably represents Clr3-myc in the SHREC complex. It should be noted that bulk H3 was detected in fractions near the void (Fig 4D); thus, it is also possible that the faster-eluted peak corresponds to Clr3-myc associated with chromatin through other SHREC components.

Clr3-myc was distributed similarly in the wild-type strain and strains lacking a SHREC component—with the exception of the *clr1*Δ strain, in which the signal intensity decreased dramatically in fractions 11–14 (Fig 4A, *clr1*Δ). This finding is consistent with reports that Clr1 is important for the integrity of the SHREC complex [12,14]. The Clr3-myc levels also decreased slightly in the *chp2*Δ strain in fractions 11–14 (Fig 4A, *chp2*Δ). Importantly, a similar reduction was also observed in the *chp2-W199A* strain (Fig 4A, *chp2-W199A*). These results support the idea that the faster-eluted peak includes chromatin-associated Clr3-myc, and suggest that Clr3’s chromatin targeting depends in part on Chp2’s H3K9me binding.

### Clr2 targets chromatin independently of Chp2’s H3K9me binding

Clr2, which has several functional domains, may serve to tether the HDAC module of the SHREC to chromatin [14]. In gel-filtration, V5-Clr2 was widely distributed across fractions in the wild-type strain (Fig 4B, wt) but was clearly concentrated in fractions 11–22 and 26–30, with peaks in fractions 11–14, 16–18, and 27–29. The peak in fractions 27–29, corresponding to an estimated molecular mass of ~120 kDa appeared to be free V5-Clr2, although V5-Clr2’s expected molecular mass is 66 kDa. Indeed, specific single-point amino acid substitutions in Clr2 negatively affected the transcriptional silencing [22] and significantly distorted this peak or removed it altogether (S2B Fig); this was presumably because the point mutations changed Clr2’s conformation and consequently its elution profiles. Interestingly, these point mutations did not affect Clr2’s own localization to fractions with higher molecular weights (S2B Fig), but slightly inhibited Clr3-myc’s co-fractionation with bulk histone H3 (S2A Fig) even though no such effect was observed for the *clr2*Δ strain (S2B Fig).

As observed for Clr3-myc, the faster-eluting V5-Clr2 in fractions 11–14 was probably a V5-Clr2 subpopulation associated with the whole SHREC complex or with chromatin, or both. The V5-Clr2 distribution was not affected by the lack of Mit1 or Chp2 or by the Chp2-W199A mutation, suggesting that Clr2 targets chromatin independently of Chp2’s H3K9me binding. Interestingly, V5-Clr2’s distribution pattern changed in the *clr1*Δ and *clr3*Δ mutant strains (Fig 4B). In these strains, V5-Clr2 was still present in the high molecular-weight fractions (fractions 11–14) and in fractions 27–29, but was significantly reduced in fractions 15–21. This finding suggests that the peak in fractions 16–18 might correspond to a subcomplex of V5-Clr2 with Clr1 and/or Clr3-myc. Although Clr3-myc’s slower-eluted peak (Fig 4A, fractions 16–19) largely overlapped with this V5-Clr2 peak, Clr3-myc’s distribution was not noticeably changed in *clr1*Δ or *clr2*Δ cells (Fig 4A). Thus, V5-Clr2’s peak in fractions 16–18 might correspond to a complex involving V5-Clr2 and Clr1, and Clr3 might indirectly affect the stability of this V5-Clr2–Clr1 complex.

Clr2 possesses an MDB-like domain, and the Clr1–Clr2 complex binds RNA and DNA through this domain [14]. Although this activity of Clr2 is thought to tether SHREC to chromatin, it might also help SHREC interact with cellular RNAs. To investigate whether SHREC contains an RNA component, or whether SHREC’s association with chromatin is dependent on RNA, native lysates were treated with RNaseA prior to chromatographic separation (S3 Fig). However, the distribution patterns of Clr3-myc, V5-Clr2 and F-Chp2 upon RNase A treatment did not change compared to the untreated control, indicating that RNA is not critical for the integrity of the SHREC complex (S3 Fig).

We used the same chromatographic approach to investigate the elution profile of F-Chp2 (Fig 4C). F-Chp2 was primarily detected in fractions 11–13 and 17–23. The faster-eluted peak overlapped with the H3 peak and the faster-eluted peaks of Clr3-myc and V5-Clr2 (Fig 4D), and thus most likely corresponded to chromatin-associated F-Chp2. Indeed, the Chp2-W199A mutant protein was not detected in fractions 11–14 (Fig 4D, *chp2-W199A*). Again, the result confirmed that Chp2-CD’s H3K9me binding was critical for its own association with chromatin. While the estimated size of the slower-eluted peak was clearly larger than the molecular mass calculated for F-Chp2 (47.5 kDa), a previous study using recombinant proteins showed that Chp2 forms a stable dimer that is detected in fractions corresponding to a molecular mass of 300–350 kDa, presumably due to its elongated shape [10]. Based on this previous observation, we concluded that the slower-eluted peak corresponds to the free F-Chp2 dimer. The F-Chp2 distribution was not affected by the lack of Mit1 or Clr2, suggesting that Chp2 targets chromatin independently of Mit1 and Clr2.

**Fig 5:**
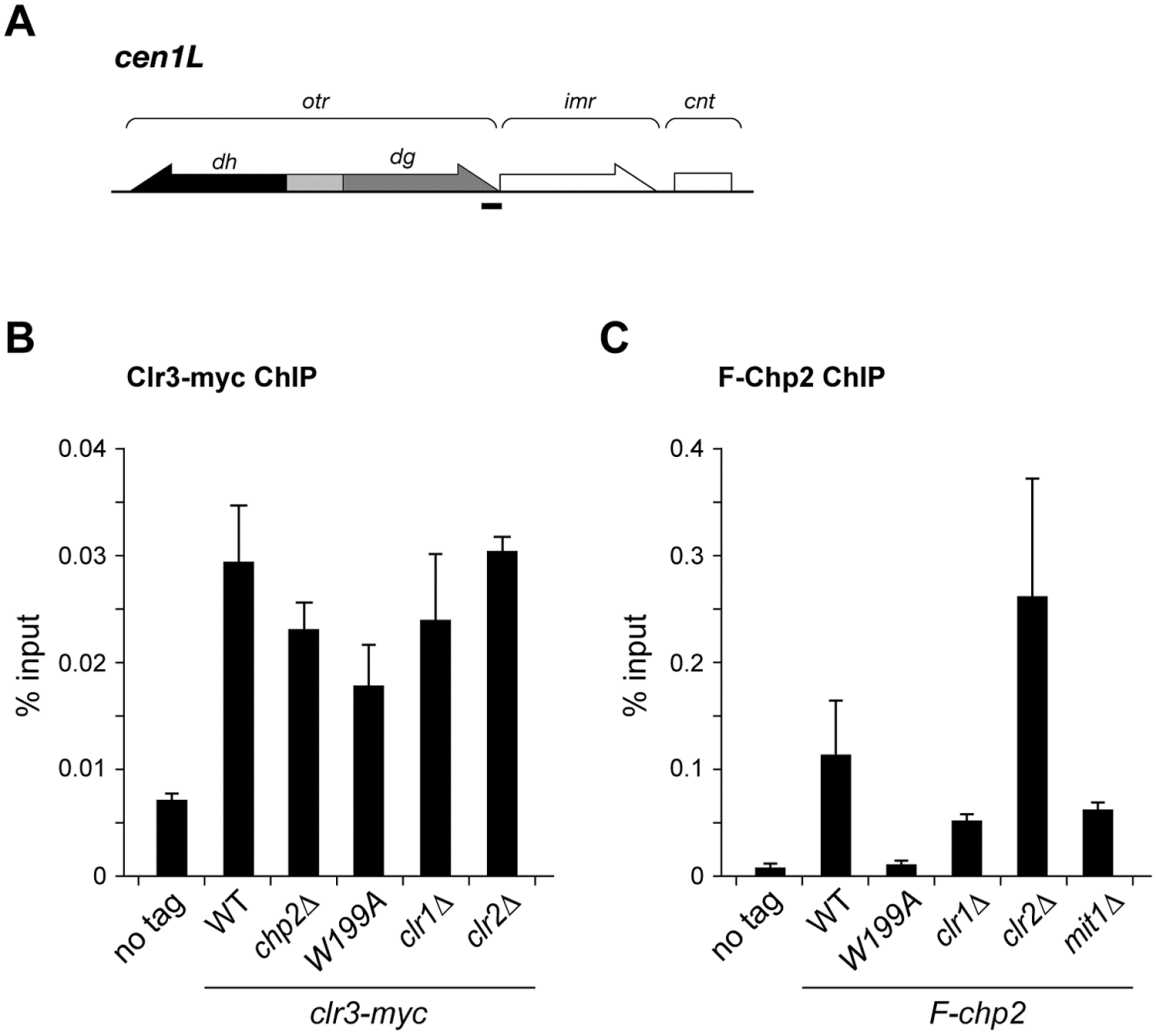
Interdependency of SHREC components to associate with heterochromatin. (A) Schematic diagram of the left half of centromere 1. Position of the PCR product used to detect Clr3-myc in (B) and F-Chp2 in (C) is indicated by a thick bar. (B,C) ChIP assay showing Clr3-myc (B) or F-Chp2 (C) levels at centromeric *dg*. The values were normalized to input. Error bars indicate standard errors from three biological replicates. The strains used were PJ1323 (wt), PJ 1600 (*clr3-myc*), EO1001 (*clr3-myc*, *chp2*Δ), EO1002 (*clr3-myc*, *F-chp2-W199*), EO1003 (*clr3-myc*, *clr1*Δ), EO1004 (*clr3-myc*, *clr2*Δ), PJ1566 (*F-chp2*), EO1005 (*F-chp2-W199A*), EO1006 (*F-chp2*, *clr1*Δ), EO1007 (*F-chp2*, *clr1*Δ), and EO1006 (*F-chp2*, *mit1*Δ).

To further confirm the relationship between Chp2’s H3K9me binding and Clr3’s chromatin targeting, we conducted ChIP assays with primers against centromeric *dg* repeats (Fig 5A), where Clr3’s preferential binding was previously observed [13]. The strain expressing Clr3-myc was enriched at the *dg* locus, whereas the enrichment was partially decreased by the lack of Chp2 or by the Chp2-W199A mutation, but not by Clr2 depletion (Fig 5B), which is well consistent with the results obtained by gel-filtration chromatography (Fig 4A). Using the same primer sets, Chp2’s centromeric *dg* binding was also examined. As observed for the *mat3-M*::*ura4*^+^ region (Fig 2E), F-Chp2’s chromatin association was compromised by Chp2-W199A mutation (Fig 5C). Although F-Chp2’s distribution was not noticeably affected in gel-filtration chromatography (Fig 4C), the enrichment was moderately decreased by the lack of Clr1 or Mit1 (Fig 5C, *clr1*Δ and *mit1*Δ), suggesting that the SHREC’s remodeling module may facilitate Chp2’s H3K9me binding. Interestingly, F-Chp2’s chromatin association was increased in *clr2*Δ cells (Fig 5C, *clr2*Δ). This result may suggest that Chp2 and Clr2 competes with each other for binding to H3K9me-enriched heterochromatic regions.

### The Chp2 protein’s stability depends on Clr3’s HDAC activity

**Fig 6:**
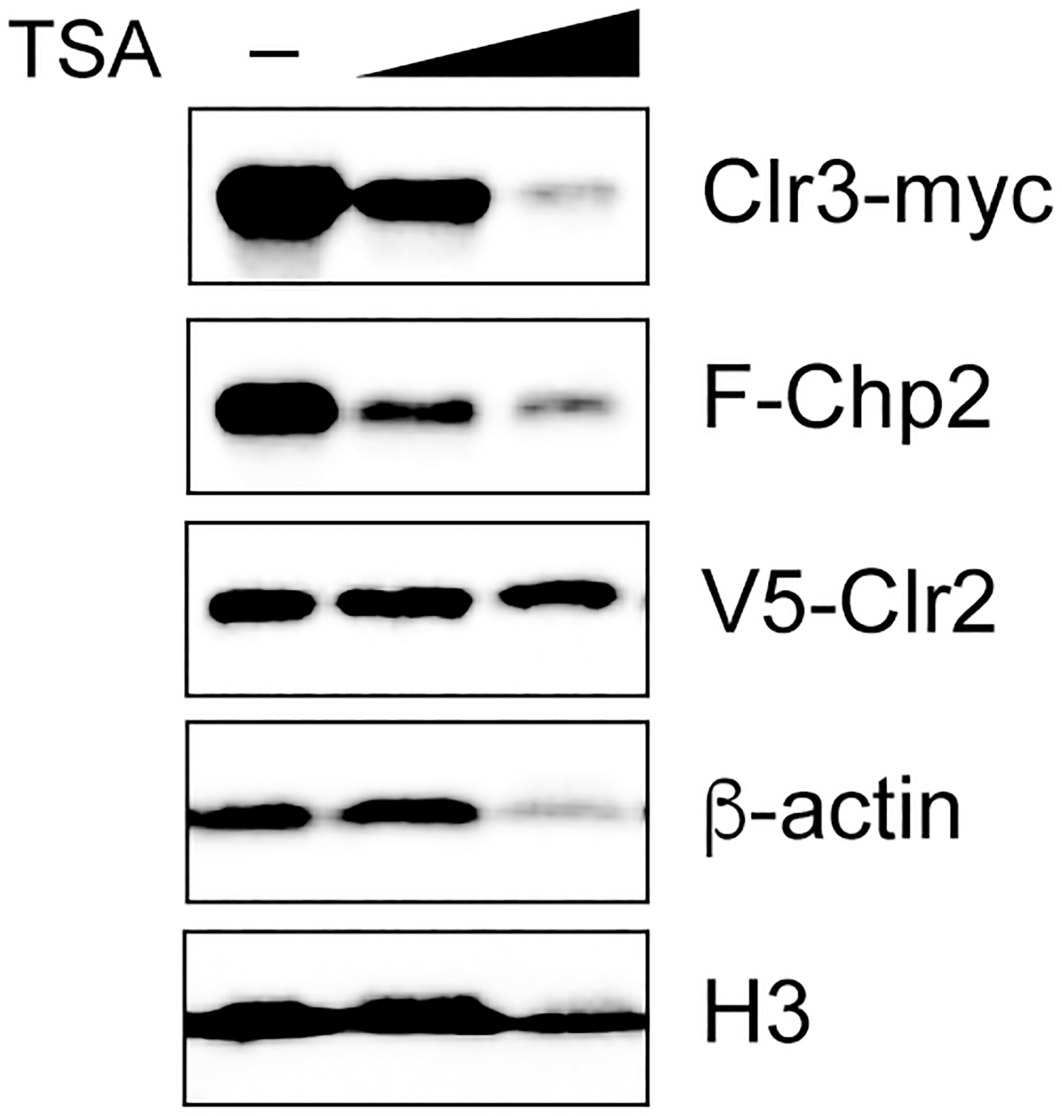
The Chp2’s protein stability depended on Clr3’s HDAC activity. Cells were grown in the presence of 1% ethanol, or with 5 or 10 μg/ml TSA. Protein levels of Clr3-myc, F-Chp2, V5-Clr2, ß-actin, and histone H3 in the whole-cell lysates were analyzed by western blotting. The strain used was PJ1911 (wt, *V5-clr2 clr3-myc F-chp2*).

In the course of experiments detecting Chp2 proteins, we found that Chp2 protein levels were partially decreased in a strain lacking Clr3 (S1B Fig). To test whether Chp2’s stability depends on Clr3’s presence or its enzymatic activity, we examined the effect of trichostatin A (TSA) treatment (Fig 6). Clr3 is a class II histone deacetylase that is inhibited by TSA [7,26]. A yeast strain expressing Clr3-myc, V5-Clr2, and F-Chp2, was treated with increasing concentrations of TSA during the growth period, resulting in a gradual reduction in F-Chp2 (Fig 6; F-Chp2). Interestingly, TSA treatment also gradually decreased and degraded Clr3-myc (Fig 6; Clr3-myc). Histone H3 was slightly reduced in a TSA-treated wild-type strain and a strain lacking Clr3, which is consistent with previous reports [11]. Although the ß-actin level was also somewhat reduced, which could indicate that TSA treatment decreased the protein levels in general, there was no significant change in the V5-Clr2 level (Fig 6). These results suggested that the Chp2 protein’s stability depends strongly on Clr3’s enzymatic HDAC activity.

## Discussion

### Chp2’s ability to bind H3K9me is crucial for its silencing function

ITC revealed that Chp2-CD bound the H3K9me3 peptide (Fig 1C) with an affinity similar to a previously reported range [10]. Chp2 might bind H3K9me-containing nucleosomes *in vivo* more strongly than might be expected from our ITC experiment, since Swi6’s affinity for chromatin increases if it forms an oligomer or is phosphorylated [27,28]. Our genetic approaches demonstrated that Chp2’s H3K9me-binding activity is essential for Chp2’s function in heterochromatin assembly (Figs 2 and 3). Interestingly, both *chp2*Δ and *chp2-W199A* mutants had an unstable silencing phenotype, with the expression of reporter genes in the mating-type region switching on and off (Fig 2B). However, the off state was incomplete, with some degree of derepression, and the cells produced pink colonies instead of the red colonies seen for the wild-type strain when grown on plates with low adenine levels (Fig 2B). There are other mutations that produce a similar switch between different epigenetic states of the mating-type region; this observation agrees with the self-propagating model of heterochromatin formation [16,22,29–31].

### Biochemical fractionation supports a two-module model for targeting SHREC to chromatin

Biochemical analysis of three SHREC components allowed us to determine which of these components are important for SHREC’s integrity and chromatin targeting. All three SHREC components analyzed in this study were cofractionated in the same fractions as histone H3, which was eluted in the fractions with higher molecular mass (Figs 4 and 5). These findings are consistent with the previously demonstrated association between all of the SHREC components and chromatin [7,13]. In addition, the failure of a SHREC component to cofractionate with histone H3 indicated a loss of association to chromatin. Our data showed that Clr3-myc was lost from high molecular-weight fractions in a *clr1*Δ strain, indicating that Clr1 is necessary for Clr3’s association with chromatin, the stability of the SHREC complex, or both. This finding is consistent with reports that Clr1 is the platform upon which the other SHREC components assemble [12,14].

Clr2 was detected in chromatin-enriched fractions, and this elution pattern was not changed by deleting any other SHREC components, indicating that Clr2 can target chromatin independently of other SHREC components (Fig 4B). Biochemical fractionation also revealed V5-Clr2 peaks in fractions 16–19 and 27–29. The slower-eluted peak appeared to correspond to free V5-Clr2, whereas the faster-eluted peak corresponded to an estimated molecular mass of about 500 kDa, probably representing a subcomplex containing V5-Clr2. The decreased V5-Clr2 signal in *clr1*Δ or *clr3*Δ cells would seem to indicate that this subcomplex contains Clr1 and Clr3 in addition to V5-Clr2. However, Clr3-myc was also detected in fractions 16–19, and this elution profile did not change noticeably in *clr1*Δ or *clr2*Δ cells (Fig 4A). Thus, it seems likely that the subcomplex consists of V5-Clr2 and Clr1, whereas Clr3 might indirectly affect the stability of this subcomplex. The reduced association of Clr3-myc with high molecular weight fractions in the strains carrying point mutations in *clr2*, but not in the strains completely lacking Clr2 (S2A Fig) was surprising. It might indicate a compensatory function of Chp2 in the targeting of Clr3 (SHREC) when Clr2 is lacking. A more severe phenotype of a point mutation as compared to a complete deletion is not unprecedented [32].

In *chp2*Δ cells or cells carrying the *chp2-W199A* mutation, the Clr3-myc signal diminished substantially in high molecular-weight fractions, indicating that either Clr3’s association with chromatin or SHREC’s stability (or both) depend partially on Chp2’s H3K9me binding (Fig 4). As with the silencing assays (Figs 2 and 3), a *chp2* deletion or *chp2-W199A* mutation had a very similar effect on Clr3-myc’s elution profile (Figs 4 and 5), supporting the conclusion that Chp2’s function relies on its ability to bind H3K9me via its CD.

Another interesting observation was that the Chp2 protein level decreased dramatically in *clr3*Δ cells, indicating that Chp2 was destabilized in the absence of Clr3 (S1B Fig). Further investigation demonstrated that treating yeast cells with TSA, which inhibits class I and class II HDAC activity, led to the degradation of Chp2 and of Clr3 itself, strongly suggesting that Clr3’s HDAC activity was involved in Chp2’s targeting and stability and in the stability of Clr3 itself (Fig 6).

In conclusion, data presented here along with previously published work suggest a two-step mechanism for SHREC’s association with chromatin, in which Clr2 binds chromatin to form a platform for recruiting Clr1 and Clr3, while Chp2 may recruit Mit1 and additionally bind and stabilize the SHREC complex at the site (Figs 4 and 5) [14]. This mechanism means that if Chp2 or Clr2 are missing, an incomplete, partially functional complex will form. This scenario agrees with an idea proposed by Job et al., that the SHREC complex functions as two separable modules, one a remodeling module containing Mit1 and Chp2, and the other an HDAC module comprised of Clr2 and Clr3 and held together by Clr1 [14]. The two are targeted independently to chromatin: the remodeling module is targeted via Chp2 and the HDAC module via Clr2, which has been shown to bind RNA and DNA [14]. However, it is well established that all of the components of the SHREC complex are required for heterochromatin formation; this is evidenced also by the minimal silencing system described in this study, in which the reporter gene was not silenced if any of the SHREC proteins were absent (Fig 3) [12,13]. Moreover, Clr3’s HDAC activity greatly affected Chp2’s targeting or stability, demonstrating the functional dependency of the two modules within the SHREC complex (Fig 6 and S1B Fig).

## Supporting information

S1 Fig Western blots of Chp2 proteins. (A) Whole-cell lysates prepared from the SPYB106 (wt), SPYB148 (*chp2*Δ), SPM2238 (*F-chp2*), and SPM2291 (*F-chp2-W199A*) strains were separated by SDS-PAGE and analyzed by western blotting with antibodies against the FLAG tag (top) or against Chp2 (middle). After western blotting, the membrane was stained by Amido black to assess the amount of loaded proteins (bottom). Asterisk: nonspecific protein band; arrowhead: endogenous Chp2 band. (B) Whole-cell lysates prepared from the PJ78 (wt), SPAH101 (*clr3*Δ #1), and SPAH102 (*clr3*Δ #2) strains were separated by SDS-PAGE and analyzed by western blotting with antibodies against Chp2 (top). After western blotting, the membrane was stained by Amido black to assess the amount of loaded proteins (bottom).
(TIF)

S2 Fig Critical amino acid substitutions in Clr2 affect Clr3’s chromatin association. (A, B) Native whole-cell lysates prepared from strains expressing Clr3-myc and V5-tagged wild-type or mutant Clr2 were fractionated by gel-filtration chromatography. Proteins from the collected fractions were precipitated and analyzed by 8% SDS-PAGE; elution profiles of Clr3-myc (A) and V5-Clr2 (B) were analyzed by western blotting. The first band in each panel represents the 1% input from yeast lysates (In). The strains used were PJ1794 (*V5-clr2 clr3-my*c), PJ1724 (*V5-clr2-Y140G clr3-myc F-chp2*), PJ1571 (*V5-clr2-R170G clr3-myc F-chp2*), PJ1727 (*V5-clr2-E376G clr3-myc F-chp2*), PJ1994 (*chp2*Δ *clr3-myc F-chp2*), and PJ1917 (*V5-clr2 clr3-myc FLAG-chp2W199A*).
(TIF)

S3 Fig RNase A treatment did not affect the SHREC-complex integrity. (A–D) Native whole-cell lysates prepared from strains expressing Clr3-myc, V5-Clr2, and F-Chp2, untreated or treated with RNase A, were fractionated by gel-filtration chromatography. Proteins from collected fractions were precipitated and analyzed by 8% SDS-PAGE. Elution profiles of Clr3-myc (A), V5-Clr2 (B), F-Chp2 (C), or bulk H3 (D) were analyzed by western blots. The first band in each panel represents the 1% input from yeast lysates (In).
(TIF)

S1 Table List of *S*. *pombe* strains used in this study. (DOCX)

## References

[1] Soshnev AA, Josefowicz SZ, Allis CD. Greater Than the Sum of Parts: Complexity of the Dynamic Epigenome. Mol Cell. 2016;62(5):681–94. 10.1016/j.molcel.2016.05.004 27259201 PMC4898265

[2] Barres R, Zierath JR. The role of diet and exercise in the transgenerational epigenetic landscape of T2DM. Nat Rev Endocrinol. 2016;12(8):441–51. 10.1038/nrendo.2016.87 27312865

[3] Luger K, Mader AW, Richmond RK, Sargent DF, Richmond TJ. Crystal structure of the nucleosome core particle at 2.8 A resolution. Nature. 1997;389(6648):251–60. 10.1038/38444 9305837

[4] Olsson I, Bjerling P. Advancing our understanding of functional genome organisation through studies in the fission yeast. Curr Genet. 2011;57(1):1–12. 10.1007/s00294-010-0327-x 21113595 PMC3023017

[5] Rodriguez A, Bjerling P. The links between chromatin spatial organization and biological function. Biochem Soc Trans. 2013;41(6):1634–9. 10.1042/BST20130213 24256267 PMC3836414

[6] Nishibuchi G, Nakayama J. Biochemical and structural properties of heterochromatin protein 1: understanding its role in chromatin assembly. J Biochem. 2014;156(1):11–20. 10.1093/jb/mvu032 24825911

[7] Bjerling P, Silverstein RA, Thon G, Caudy A, Grewal S, Ekwall K. Functional divergence between histone deacetylases in fission yeast by distinct cellular localization and in vivo specificity. Mol Cell Biol. 2002;22(7):2170–81. 10.1128/MCB.22.7.2170-2181.2002 11884604 PMC133699

[8] Buscaino A, Lejeune E, Audergon P, Hamilton G, Pidoux A, Allshire RC. Distinct roles for Sir2 and RNAi in centromeric heterochromatin nucleation, spreading and maintenance. EMBO J. 2013;32(9):1250–64. 10.1038/emboj.2013.72 23572080 PMC3642681

[9] Bannister AJ, Zegerman P, Partridge JF, Miska EA, Thomas JO, Allshire RC, et al Selective recognition of methylated lysine 9 on histone H3 by the HP1 chromo domain. Nature. 2001;410(6824):120–4. 10.1038/35065138 11242054

[10] Sadaie M, Kawaguchi R, Ohtani Y, Arisaka F, Tanaka K, Shirahige K, et al Balance between distinct HP1 family proteins controls heterochromatin assembly in fission yeast. Mol Cell Biol. 2008;28(23):6973–88. 10.1128/MCB.00791-08 18809570 PMC2593388

[11] Wiren M, Silverstein RA, Sinha I, Walfridsson J, Lee HM, Laurenson P, et al Genomewide analysis of nucleosome density histone acetylation and HDAC function in fission yeast. EMBO J. 2005;24(16):2906–18. 10.1038/sj.emboj.7600758 16079916 PMC1187943

[12] Motamedi MR, Hong EJ, Li X, Gerber S, Denison C, Gygi S, et al HP1 proteins form distinct complexes and mediate heterochromatic gene silencing by nonoverlapping mechanisms. Mol Cell. 2008;32(6):778–90. Epub 2008/12/30. 10.1016/j.molcel.2008.10.026 19111658 PMC2735125

[13] Sugiyama T, Cam HP, Sugiyama R, Noma K, Zofall M, Kobayashi R, et al SHREC, an effector complex for heterochromatic transcriptional silencing. Cell. 2007;128(3):491–504. Epub 2007/02/10. 10.1016/j.cell.2006.12.035 17289569

[14] Job G, Brugger C, Xu T, Lowe BR, Pfister Y, Qu C, et al SHREC Silences Heterochromatin via Distinct Remodeling and Deacetylation Modules. Mol Cell. 2016;62(2):207–21. 10.1016/j.molcel.2016.03.016 27105116 PMC4890606

[15] Sawano A, Miyawaki A. Directed evolution of green fluorescent protein by a new versatile PCR strategy for site-directed and semi-random mutagenesis. Nucleic Acids Res. 2000;28(16):E78 10931937 10.1093/nar/28.16.e78 PMC108465

[16] Thon G, Friis T. Epigenetic inheritance of transcriptional silencing and switching competence in fission yeast. Genetics. 1997;145(3):685–96. 9055078 10.1093/genetics/145.3.685 PMC1207853

[17] Bayne EH, White SA, Kagansky A, Bijos DA, Sanchez-Pulido L, Hoe KL, et al Stc1: a critical link between RNAi and chromatin modification required for heterochromatin integrity. Cell. 2010;140(5):666–77. Epub 2010/03/10. 10.1016/j.cell.2010.01.038 20211136 PMC2875855

[18] Simon P. Q-Gene: processing quantitative real-time RT-PCR data. Bioinformatics. 2003;19(11):1439–40. 12874059 10.1093/bioinformatics/btg157

[19] Kurdistani SK, Robyr D, Tavazoie S, Grunstein M. Genome-wide binding map of the histone deacetylase Rpd3 in yeast. Nat Genet. 2002;31(3):248–54. 10.1038/ng907 12089521

[20] Robyr D, Grunstein M. Genomewide histone acetylation microarrays. Methods. 2003;31(1):83–9. 12893177 10.1016/s1046-2023(03)00091-4

[21] Boeke JD, Trueheart J, Natsoulis G, Fink GR. 5-Fluoroorotic acid as a selective agent in yeast molecular genetics. Methods Enzymol. 1987;154:164–75. 3323810 10.1016/0076-6879(87)54076-9

[22] Steinhauf D, Rodriguez A, Vlachakis D, Virgo G, Maksimov V, Kristell C, et al Silencing motifs in the Clr2 protein from fission yeast, Schizosaccharomyces pombe. PLoS One. 2014;9(1):e86948 10.1371/journal.pone.0086948 24475199 PMC3903592

[23] Smirnov MN, Smirnov VN, Budowsky EI, Inge-Vechtomov SG, Serebrjakov NG. Red pigment of adenine-deficient yeast Saccharomyces cerevisiae. Biochem Biophys Res Commun. 1967;27(3):299–304. 6035110 10.1016/s0006-291x(67)80096-2

[24] Kagansky A, Folco HD, Almeida R, Pidoux AL, Boukaba A, Simmer F, et al Synthetic heterochromatin bypasses RNAi and centromeric repeats to establish functional centromeres. Science. 2009;324(5935):1716–9. 10.1126/science.1172026 19556509 PMC2949999

[25] Yamada T, Fischle W, Sugiyama T, Allis CD, Grewal SI. The nucleation and maintenance of heterochromatin by a histone deacetylase in fission yeast. Mol Cell. 2005;20(2):173–85. 10.1016/j.molcel.2005.10.002 16246721

[26] Vanhaecke T, Papeleu P, Elaut G, Rogiers V. Trichostatin A-like hydroxamate histone deacetylase inhibitors as therapeutic agents: toxicological point of view. Curr Med Chem. 2004;11(12):1629–43. 15180568 10.2174/0929867043365099

[27] Canzio D, Chang EY, Shankar S, Kuchenbecker KM, Simon MD, Madhani HD, et al Chromodomain-mediated oligomerization of HP1 suggests a nucleosome-bridging mechanism for heterochromatin assembly. Mol Cell. 2011;41(1):67–81. 10.1016/j.molcel.2010.12.016 21211724 PMC3752404

[28] Nishibuchi G, Machida S, Osakabe A, Murakoshi H, Hiragami-Hamada K, Nakagawa R, et al N-terminal phosphorylation of HP1alpha increases its nucleosome-binding specificity. Nucleic Acids Res. 2014;42(20):12498–511. 10.1093/nar/gku995 25332400 PMC4227797

[29] Grewal SI, Klar AJ. Chromosomal inheritance of epigenetic states in fission yeast during mitosis and meiosis. Cell. 1996;86(1):95–101. 8689692 10.1016/s0092-8674(00)80080-x

[30] Zhang K, Mosch K, Fischle W, Grewal SI. Roles of the Clr4 methyltransferase complex in nucleation, spreading and maintenance of heterochromatin. Nat Struct Mol Biol. 2008;15(4):381–8. 10.1038/nsmb.1406 18345014

[31] Al-Sady B, Madhani HD, Narlikar GJ. Division of labor between the chromodomains of HP1 and Suv39 methylase enables coordination of heterochromatin spread. Mol Cell. 2013;51(1):80–91. 10.1016/j.molcel.2013.06.013 23849629 PMC3752401

[32] Hansen KR, Burns G, Mata J, Volpe TA, Martienssen RA, Bahler J, et al Global effects on gene expression in fission yeast by silencing and RNA interference machineries. Mol Cell Biol. 2005;25(2):590–601. 10.1128/MCB.25.2.590-601.2005 15632061 PMC543407

