# Supplementary figures and images for: The binding of Chp2’s chromodomain to methylated H3K9 is essential for Chp2’s role in heterochromatin assembly in fission yeast
Source: PLoS One. 2018 Aug 15;13(8):e0201101. doi: 10.1371/journal.pone.0201101 (PMC6093649; doi:10.1371/journal.pone.0201101)

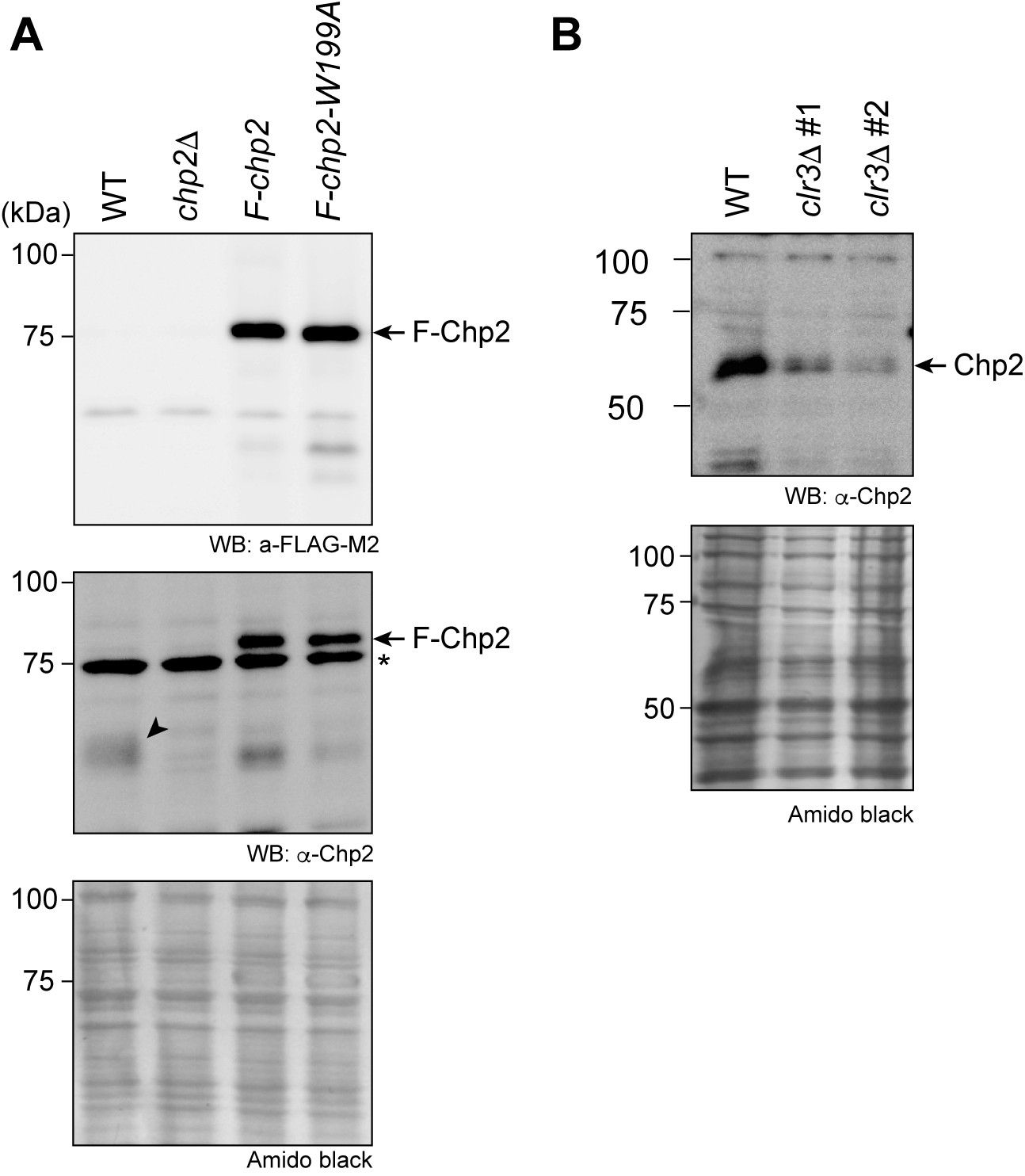

Supplement: S1 Fig — (A) Whole-cell lysates prepared from the SPYB106 (wt), SPYB148 (chp2Δ), SPM2238 (F-chp2), and SPM2291 (F-chp2-W199A) strains were separated by SDS-PAGE and analyzed by western blotting with antibodies against the FLAG tag (top) or against Chp2 (middle). After western blotting, the membrane was stained by Amido black to assess the amount of loaded proteins (bottom). Asterisk: nonspecific protein band; arrowhead: endogenous Chp2 band. (B) Whole-cell lysates prepared from the PJ78 (wt), SPAH101 (clr3Δ #1), and SPAH102 (clr3Δ #2) strains were separated by SDS-PAGE and analyzed by western blotting with antibodies against Chp2 (top). After western blotting, the membrane was stained by Amido black to assess the amount of loaded proteins (bottom). (TIF) [file pone.0201101.s001.tif]

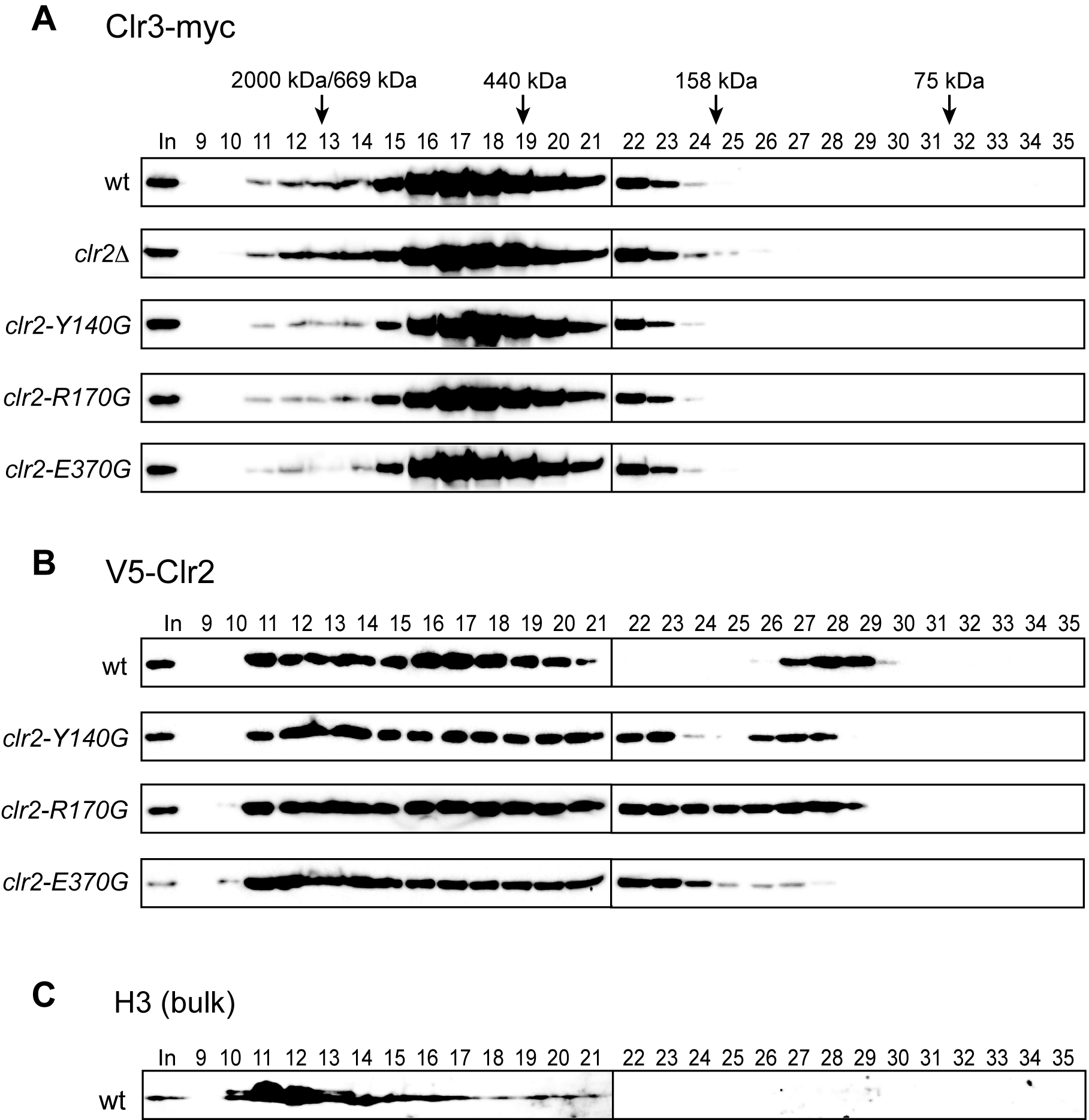

Supplement: S2 Fig — (A, B) Native whole-cell lysates prepared from strains expressing Clr3-myc and V5-tagged wild-type or mutant Clr2 were fractionated by gel-filtration chromatography. Proteins from the collected fractions were precipitated and analyzed by 8% SDS-PAGE; elution profiles of Clr3-myc (A) and V5-Clr2 (B) were analyzed by western blotting. The first band in each panel represents the 1% input from yeast lysates (In). The strains used were PJ1794 (V5-clr2 clr3-myc), PJ1724 (V5-clr2-Y140G clr3-myc F-chp2), PJ1571 (V5-clr2-R170G clr3-myc F-chp2), PJ1727 (V5-clr2-E376G clr3-myc F-chp2), PJ1994 (chp2Δ clr3-myc F-chp2), and PJ1917 (V5-clr2 clr3-myc FLAG-chp2W199A). (TIF) [file pone.0201101.s002.tif]

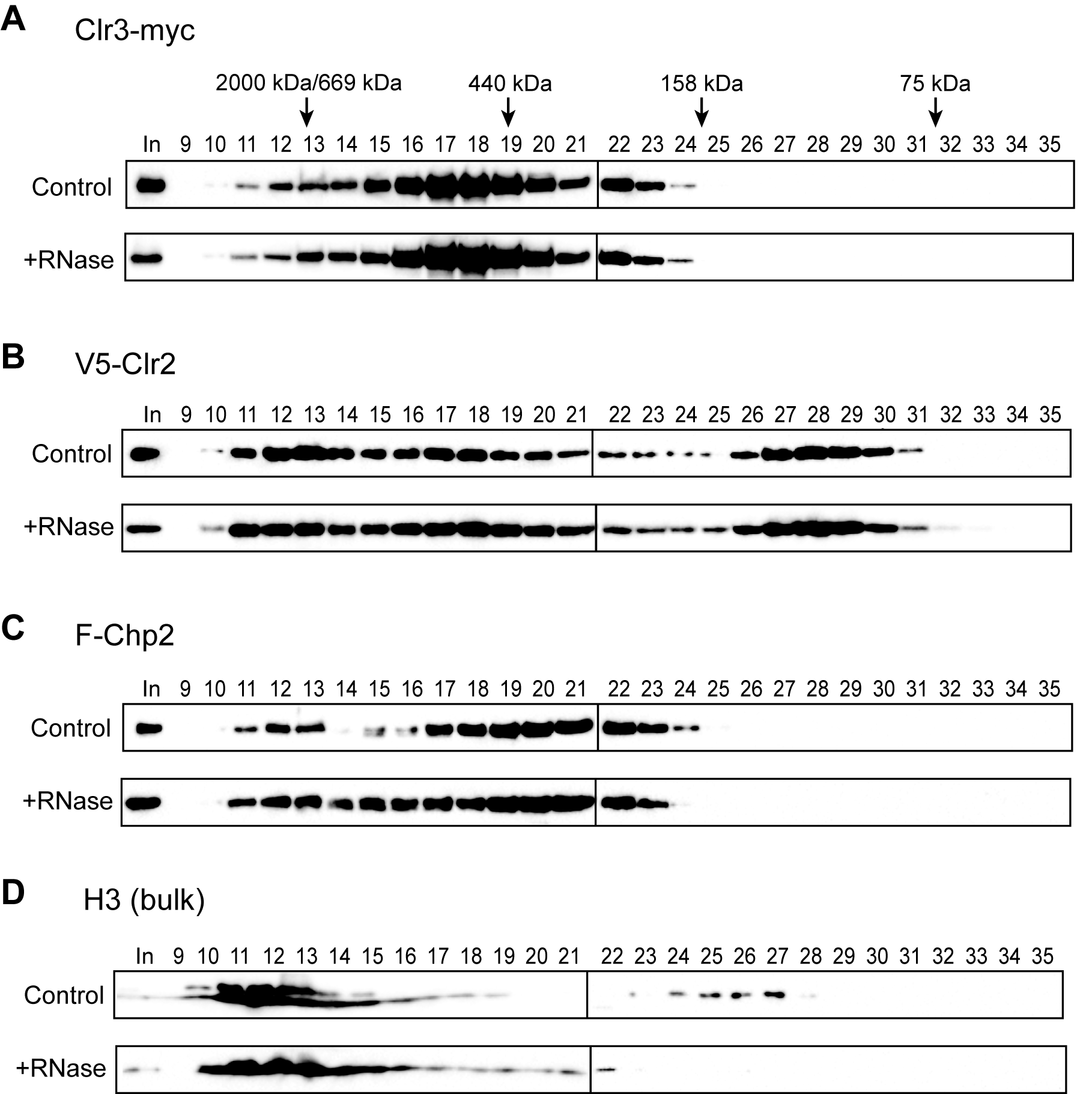

Supplement: S3 Fig — (A–D) Native whole-cell lysates prepared from strains expressing Clr3-myc, V5-Clr2, and F-Chp2, untreated or treated with RNase A, were fractionated by gel-filtration chromatography. Proteins from collected fractions were precipitated and analyzed by 8% SDS-PAGE. Elution profiles of Clr3-myc (A), V5-Clr2 (B), F-Chp2 (C), or bulk H3 (D) were analyzed by western blots. The first band in each panel represents the 1% input from yeast lysates (In). (TIF) [file pone.0201101.s003.tif]
