# Supplementary material for: The binding of Chp2’s chromodomain to methylated H3K9 is essential for Chp2’s role in heterochromatin assembly in fission yeast
Source: PLoS One. 2018 Aug 15;13(8):e0201101. doi: 10.1371/journal.pone.0201101 (PMC6093649; doi:10.1371/journal.pone.0201101)
Supplement: S1 Table — (DOCX) [file pone.0201101.s004.docx]

**S1 Table. List of *S. pombe* strains used in this study.**

| Strain | Genotype | Source |
| --- | --- | --- |
| PJ1811 | *h^90^ clr3-myc::kanMX6 mat3-M::ura4^+^ ura4-DS/E//D18 leu1-32 ade6-M210* | This study |
| PJ1813 | *h^90^ clr3-myc::kanMX6 chp2*Δ*::LEU2^+^ mat3-M::ura4^+^ ura4-DS/E//D18 leu1-32 ade6-M210* | This study |
| PJ1815 | *h^90^ clr3-myc::kanMX6 Flag-chp2 mat3-M::ura4^+^ ura4-DS/E//D18 leu1-32 ade6-M210* | This study |
| PJ1817 | *h^90^ clr3-myc::kanMX6 Flag-chp2-W199A mat3-M::ura4^+^ leu1-32 ura4-DS/E//D18 ade6-M210//DN/N* (#1) | This study |
| PJ1818 | *h^90^ clr3-myc::kanMX6 Flag-chp2-W199A mat3-M::ura4^+^ leu1-32 ura4-DS/E//D18 ade6-M210//DN/N* (#2) | This study |
| PJ1819 | *h^90^ clr3-myc::kanMX6 Flag-chp2-W199A mat3-M::ura4^+^ leu1-32 ura4-DS/E//D18 ade6-M210//DN/N* (#3) | This study |
| PJ1044 | *h^90^ mat3-M::ade6^+^ leu1-32 ura4-D18 ade6-DN/N* | Steinhauf (2014) |
| PJ1569 | *h^90^ chp2*Δ*::LEU2^+^ mat3-M::ade6^+^ leu1-32 ura4-D18 ade6-DN/N* | This study |
| PJ1770 | *h^90^ Flag-chp2 mat3-M::ade6^+^ leu1-32 ura4- D18 ade6-DN/N* | This study |
| PJ1538 | *h^90^ Flag-chp2-W199A mat3-M::ade6^+^ leu1-32 ura4-D18 ade6-DN/N* | This study |
| FY597 | *h^90^ mat3-M::ura4^+^ ura4-DS/E leu1-32 ade6-M210* | Allshire (1995) |
| PJ748 | *h^90^ mat3-M::ura4^+^ chp2*Δ*::LEU2^+^ ura4-DS/E leu1-32 ade6-M210* | This study |
| PJ1745 | *h^90^ mat3-M::ura4^+^ Flag-chp2 ura4-DS/E leu1-32 ade6-M210//DN/N* | This study |
| PJ1735 | *h^90^ mat3-M::ura4^+^ Flag-chp2-W199A ura4-DS/E leu1-32 ade6-M210//DN/N* (#1) | This study |
| PJ1736 | *h^90^ mat3-M::ura4^+^ Flag-chp2-W199A ura4-DS/E leu1-32 ade6-M210//DN/N* (#2) | This study |
| PJ1737 | *h^90^ mat3-M::ura4^+^ Flag-chp2-W199A ura4-DS/E leu1-32 ade6-M210//DN/N* (#3) | This study |
| PJ1207 | *h^+^ ura4::3gbs-ade6^+^ leu1-32 ade6-DN/N* | Steinhauf (2014) |
| PJ1231 | *h^+^ GBD-clr4*Δ*CD::hphMX6 ura4::3gbs-ade6^+^ leu1-32 ade6-DN/N* | Steinhauf (2014) |
| PJ1327 | *h^+^ clr2∆::ura4^+^ GBD-clr4*Δ*CD::hphMX6 ura4::3gbs-ade6^+^ leu1-32 ade6-DN/N* | Steinhauf (2014) |
| PJ1246 | *h^+^ clr1∆::LEU2^+^ GBD-clr4*Δ*CD::hphMX6 ura4::3gbs-ade6^+^ leu1-32 ade6-DN/N* | This study |
| PJ1247 | *h^+^ clr3∆::kanMX6 GBD-clr4*Δ*CD::hphMX6 ura4::3gbs-ade6^+^ leu1-32 ade6-DN/N* | This study |
| PJ1320 | *h^+^ mit1∆::kanMX6 GBD-clr4*Δ*CD::hphMX6 ura4::3gbs-ade6^+^ leu1-32 ade6-DN/N* | This study |
| PJ1314 | *h^+^ chp2∆::LEU2^+^ GBD-clr4*Δ*CD::hphMX6 ura4::3gbs-ade6^+^ leu1-32 ade6-DN/N* | This study |
| PJ1318 | *h^+^ GBD-clr4*Δ*CD::hphMX6 ura4::3gbs-ade6^+^ imr1R(NcoI)::ura4^+^ leu1-32 ade6-DN/N* | This study |
| PJ1316 | *h^+^ clr3-735 GBD-clr4*Δ*CD::hphMX6 ura4::3gbs-ade6^+^ imr1R(NcoI)::ura4^+^ leu1-32 ade6-DN/N* | This study |
| PJ1325 | *h^+^ F-chp2 GBD-clr4*Δ*CD::hphMX6 ura4::3gbs-ade6^+^ leu1-32 ade6-DN/N* | This study |
| PJ1331 | *h^+^ Flag-chp2-W199A GBD-clr4*Δ*CD::hphMX6 ura4::3gbs-ade6^+^ leu1-32 ade6-DN/N* | This study |
| PJ1911 | *h^-^ V5-clr2 clr3-myc::kanMX6 Flag-chp2 leu1-32 ura4-D18 ade6-M210//DN/N* | This study |
| PJ2012 | *h^-^ V5-clr2 clr3-myc::kanMX6 Flag-chp2 mit1*Δ*::kanMX6 leu1-32 ura4-D18 ade6-M210//DN/N* | This study |
| PJ1836 | *h^-^ V5-clr2 clr3-myc::kanMX6 clr1*Δ*::LEU2^+^ leu1-32 ura4-D18 ade6-M210//DN/N* | This study |
| PJ1994 | *h^-^ clr3-myc::kanMX6 Flag-chp2 clr2*Δ*::ura4^+^ leu1-32 ura4-D18 ade6-M210//DN/N* | This study |
| PJ1840 | *h^-^ V5-clr2 clr3-myc::kanMX6 chp2*Δ*::LEU2^+^ leu1-32 ura4-D18 ade6-M210//DN/N* | This study |
| PJ1917 | *h^-^ V5-clr2 clr3-myc::kanMX6 Flag-chp2-W199A leu1-32 ura4-D18 ade6-M210//DN/N* | This study |
| PJ1323 | *h^-^ leu1-32 ura4-D18 ade6-M210//DN/N* | This study |
| PJ1600 | *h^-^ clr3-myc::kanMX6 leu1-32 ura4-D18 ade6-M210//DN/N* | This study |
| EO1001 | *h^-^ chp2∆::LEU2^+^ clr3-myc::kanMX6 leu1-32 ura4-D18 ade6-M210//DN/N* | This study |
| EO1002 | *h^-^ Flag-chp2-W199A clr3-myc::kanMX6 leu1-32 ura4-D18 ade6-M210//DN/N* | This study |
| EO1003 | *h^-^ clr1*Δ*::LEU2^+^ clr3-myc::kanMX6 leu1-32 ura4-D18 ade6-M210//DN/N* | This study |
| EO1004 | *h^-^ clr2∆::ura4^+^ clr3-myc::kanMX6 leu1-32 ura4-D18 ade6-M210//DN/N* | This study |
| PJ1566 | *h^-^ Flag-chp2 leu1-32 ura4-D18 ade6-M210//DN/N* | This study |
| EO1005 | *h^-^ Flag-chp2-W199A leu1-32 ura4-D18 ade6-M210//DN/N* | This study |
| EO1006 | *h^-^ clr1*Δ*::LEU2^+^ Flag-chp2 leu1-32 ura4-D18 ade6-M210//DN/N* | This study |
| EO1007 | *h^-^ clr2∆::ura4^+^ Flag-chp2 leu1-32 ura4-D18 ade6-M210//DN/N* | This study |
| EO1008 | *h^-^ mit1*Δ*::kanMX6 Flag-chp2 leu1-32 ura4-D18 ade6-M210//DN/N* | This study |
| SPYB106 | *h^90^ leu1-32 his2 ura4-DS/E ade6-M216 Kint2::ura4^+^* | Sadaie  (2004) |
| SPYB148 | *h^90^ leu1-32 his2 ura4-DS/E ade6-M216 Kint2::ura4^+^ chp2∆::kanMX6* | Sadaie  (2004) |
| SPM2238 | *h^90^ Flag-chp2 leu1-32 his2 ura4-DS/E ade6-M216 Kint2::ura4^+^* | This study |
| SPM2291 | *h^90^ Flag-chp2-W199A leu1-32 his2 ura4-DS/E ade6-M216 Kint2::ura4^+^* | This study |
| PJ78 | *h^+^ leu1-32 ura4-D18 his7-366 ade6-DN/N* | Bjerling  (2007) |
| SPAH101 | *h^+^ chp2∆::hphMX6 leu1-32 ura4-D18 his7-366 ade6-DN/N* (#1) | This study |
| SPAH102 | *h^+^ chp2∆::hphMX6 leu1-32 ura4-D18 his7-366 ade6-DN/N* (#2) | This study |
| PJ1794 | *h^-^ V5-clr2 clr3-myc::kanMX6 leu1-32 ura4-D18 ade6-M210//DN/N* | This study |
| PJ1724 | *h^-^ V5-clr2-Y140G clr3-myc::kanMX6 leu1-32 ura4-D18 ade6-M210//DN/N* | This study |
| PJ1571 | *h^-^ V5-clr2-R170G clr3-myc::kanMX6 leu1-32 ura4-D18 ade6-M210//DN/N* | This study |
| PJ1727 | *h^-^ V5-clr2-E376G clr3-myc::kanMX6 leu1-32 ura4-D18 ade6-M210//DN/N* | This study |
|  |  |  |
